# Genetic differences between willow warbler migratory phenotypes are few and cluster in large haplotype blocks

**DOI:** 10.1002/evl3.15

**Published:** 2017-06-16

**Authors:** Max Lundberg, Miriam Liedvogel, Keith Larson, Hanna Sigeman, Mats Grahn, Anthony Wright, Susanne Åkesson, Staffan Bensch

**Affiliations:** ^1^ Department of Biology Lund University SE 22362 Lund Sweden; ^2^ Max Planck Institute for Evolutionary Biology MPRG Behavioural Genomics August‐Thienemann‐Straße 2 24306 Plön Germany; ^3^ Climate Impacts Research Centre, Department of Ecology and Environmental Sciences Umeå University SE 90187 Umeå Sweden; ^4^ School of Natural Sciences, Technology and Environmental Studies Södertörn University Huddinge SE 141 89 Sweden; ^5^ Department of Laboratory Medicine, Clinical Research Center, Karolinska Institute Karolinska University Hospital Huddinge SE 14186 Sweden

**Keywords:** Divergent chromosome region, local adaptation, migration

## Abstract

It is well established that differences in migratory behavior between populations of songbirds have a genetic basis but the actual genes underlying these traits remains largely unknown. In an attempt to identify such candidate genes we *de novo* assembled the genome of the willow warbler *Phylloscopus trochilus*, and used whole‐genome resequencing and a SNP array to associate genomic variation with migratory phenotypes across two migratory divides around the Baltic Sea that separate SW migrating *P. t. trochilus* wintering in western Africa and SSE migrating *P. t. acredula* wintering in eastern and southern Africa. We found that the genomes of the two migratory phenotypes lack clear differences except for three highly differentiated regions located on chromosomes 1, 3, and 5 (containing 146, 135, and 53 genes, respectively). Within each migratory phenotype we found virtually no differences in allele frequencies for thousands of SNPs, even when comparing geographically distant populations breeding in Scandinavia and Far East Russia (>6000 km). In each of the three differentiated regions, multidimensional scaling‐based clustering of SNP genotypes from more than 1100 individuals demonstrates the presence of distinct haplotype clusters that are associated with each migratory phenotype. In turn, this suggests that recombination is absent or rare between haplotypes, which could be explained by inversion polymorphisms. Whereas SNP alleles on chromosome 3 correlate with breeding altitude and latitude, the allele distribution within the regions on chromosomes 1 and 5 perfectly matches the geographical distribution of the migratory phenotypes. The most differentiated 10 kb windows and missense mutations within these differentiated regions are associated with genes involved in fatty acid synthesis, possibly representing physiological adaptations to the different migratory strategies. The ∼200 genes in these regions, of which several lack described function, will direct future experimental and comparative studies in the search for genes that underlie important migratory traits.

**Impact Summary**

How animals find their way when migrating between continents is one of the most fascinating phenomena in nature. It is well established that migratory behavior has a strong genetic basis in many bird species, and different routes and wintering areas are also likely to select for adaptations related to optimal migratory performance, such as changes in physiology. However, virtually nothing is known about the specific genes underlying these traits. Here we aim to detect migration genes by contrasting the genomes of two recently diverged populations of a small migratory songbird, the willow warbler, which are very similar in appearance but that differ markedly in migration routes and wintering areas in Africa. By assaying variation throughout the genomes of 18 willow warblers, and a fraction of the genome in >1100 samples, we found very few highly differentiated loci between the populations and extremely low genome‐wide differentiation even across samples collected over vast geographical distances. The few highly differentiated loci were almost exclusively localized within three large regions on chromosomes 1, 3, and 5, containing 146, 135, and 53 genes, respectively. The genetic variation in chromosome 3 was associated with breeding altitude and latitude, but not with the migratory phenotypes. The genetic variation on chromosomes 1 and 5 perfectly matched the geographical distribution of the migratory phenotype and is thus likely to contain genes important in shaping migratory traits. We find some evidence of divergent selection in genes related to fatty acid synthesis, which could represent physiological adaptations to the different migratory routes of the willow warblers. Future studies should investigate the genes within these regions in other closely related but differentially migrating bird populations. These analyses will address whether these large migration‐related genomic regions are unique to the willow warbler or whether the genes within them have a general effect on migration in birds.

## Introduction

The seasonal migration between breeding and wintering areas, and in particular those journeys across continents undertaken by various animals such as birds and insects, represents one of the most fascinating phenomena in nature. Selective breeding and displacement experiments have clearly shown that the migratory behavior in songbirds must be encoded as an innate set of migratory directions and a schedule that provides sufficient information to reach a specific wintering area (Perdeck 1958; Berthold 1990; Helbig 1996). In addition to differences in timing and direction, differences in migratory routes and wintering areas may also select for several morphological and physiological adaptations (Pulido 2007). Even though migration‐related adaptations have generally been shown to have a strong genetic component, their underlying genetic architecture remains largely unknown (Liedvogel et al. 2011). Studies of birds using candidate gene approaches or genomic scans have found associations of several genes or genomic regions with differentially migrating populations (Mueller et al. 2011; Lundberg et al. 2013; Ruegg et al. 2014; Delmore et al. 2016) but there is so far no association that has been consistently shared across studies.

In birds, seasonal long‐distance migration has evolved and been lost repeatedly over the course of evolution (Rolland et al. 2014), but as long as we have not identified the actual genes underlying migratory traits, we cannot address whether these apparent parallel changes involve the same or lineage‐specific sets of genes. Migration genes could, in principle, be identified by comparing the genomes of closely related populations that significantly differ in their migratory strategy, such as differences in migratory directions exhibited across migratory divides. In this case different adaptations in migratory traits in each of the populations would be expected to lead to excessive allele frequency differences in the underlying loci as compared to the genome on average. Such excessive differences are predicted to be easier to detect when the background differentiation is low (Crawford and Nielsen 2013).

The willow warbler *Phylloscopus trochilus* and its two subspecies offer an excellent model for exploring the genetics of migratory traits. The subspecies breeding in western Europe (*P. t. trochilus*) migrates SW to wintering areas in West Africa, while birds breeding in northern and eastern Europe (*P. t. acredula*) migrate SSE to wintering areas in Eastern and Southern Africa (Bensch et al. 2006). Plumage color and size were originally used to describe the subspecies but these traits show extensive variation and overlap between the subspecies (Bensch et al. 2009). As differences in migratory strategy clearly separate both subspecies, we will below refer to them as southern (*trochilus*) and northern (*acredula*) migratory phenotypes. Previous studies have indicated that the willow warbler migratory phenotypes are extremely similar genetically (Lundberg et al. 2011; Lundberg et al. 2013). The lack of other apparent phenotypic and ecological differences suggests that genetic differences between the migratory phenotypes are likely to be enriched for adaptations associated with their different migratory strategies.

The most comprehensive study characterizing genetic variation in the willow warbler so far used transcriptome sequencing to identify sequence differences between the migratory phenotypes (Lundberg et al. 2013). Only a small fraction of SNPs, which clustered in three regions on chromosomes 1, 3, and 5, was found to be highly differentiated between the two phenotypes. However, the low number of samples from each migratory phenotype, subsequent pooling of samples prior to sequencing and large variation in coverage between different genes made it difficult to quantify allele‐frequency differences between the populations and hence detect potential migration genes. Here, we expanded on this approach by *de novo* assembling a reference genome for the willow warbler, performing whole‐genome resequencing of nine individuals from each migratory phenotype, and by designing a 6000 SNP array based on willow warbler transcriptome and restriction‐associated DNA (RAD) sequencing to explore genetic variation in more than 1100 willow warblers from Northern Europe and Asia. This comprehensive dataset allowed us to (i) precisely delineate the location and size of the chromosome regions that contained highly differentiated loci in previous studies, (ii) identify further sequence differences between the migratory phenotypes, (iii) quantify the association of these differences with phenotypic traits, including stable isotopes ratio in feathers, which is a proxy for migratory direction, and (iv) detect structural genomic differences between the migratory phenotypes.

The combined data from whole‐genome resequencing and the SNP array demonstrated that the genomes of the migratory phenotypes were virtually undifferentiated except for three differentiated chromosome regions (4.0–13.1 Mb) that previously had been shown to contain peaks of high differentiation in the transcriptome sequencing study (Lundberg et al. 2013). Here, we show that each of the differentiated regions contained two clusters of highly divergent haplotypes, whose frequencies for the regions on chromosome 1 and 5 matched perfectly with the geographic distribution of the migratory phenotypes.

## Methods

### DE NOVO GENOME SEQUENCING AND ASSEMBLY

We created a *de novo* assembly of the willow warbler genome in order to obtain a more unbiased reference than directly relying on the zebra finch genome, which had been the case in the previous transcriptome study, and to be able to efficiently map and assay variation in whole‐genome resequencing data. For *de novo* genome sequencing we used DNA from a single northern male caught in Stordalen, Northern Sweden (68.3°N, 19.1°E) in 2001. This DNA had been extracted from blood using a phenol‐chloroform protocol (Sambrook et al. 1989). Four different libraries were prepared with targeted insert sizes of 180 bp, 650 bp, 3 kb, and 7 kb, respectively, and sequenced on a Hiseq 2500 (Illumina, CA, USA). An assembly was constructed from 147 Gb trimmed sequence data using ALLPATHS‐LG (Gnerre et al. 2011) and subsequent gap closure and contamination removal (Supplementary methods). To arrange the scaffolds in a putative order along chromosomes in the willow warbler genome, we aligned them to the genomes of the zebra finch, collared flycatcher, and chicken using SatsumaSynteny (Grabherr et al. 2010).

### ANNOTATION

A preliminary set of protein‐coding gene models was synthesized in MAKER (Holt and Yandell 2011) using gene predictions from augustus (Stanke et al. 2006) with chicken parameters and evidence from previous RNA sequence data (Lundberg et al. 2013) and protein and transcript sequences from other bird species (Supplementary methods). The gene models were functionally annotated based on homology to other vertebrate proteins or presence of known functional domains. Gene models that did not show homology to other proteins or lacked assignment to any functional domains were discarded.

### WHOLE GENOME RESEQUENCING

Nine samples from each migratory phenotype were used for whole‐genome resequencing (Table S1). For each individual we sequenced libraries with a targeted insert size of 650 bp on a Hiseq2500 (Illumina, CA, USA), and mapped the reads to the reference genome using bwa mem (Li and Durbin 2009). Following removal of read duplicates using picardtools (http://broadinstitute.github.io/picard/), we used freebayes (Garrison and Marth 2012) to call genotypes from the filtered whole‐genome resequencing data, and a combination of vcftools (Danecek et al. 2011) and bcftools (Li 2011) to filter the raw genotype dataset based on SNP quality score, overlap with annotated repeats, site, and sample coverage and missingness of genotypes per site (Supplementary methods).

### SNP ARRAY

A SNP array was designed prior to the willow warbler genome assembly to genotype 6000 SNPs in 1152 samples. These samples were primarily collected across the hybrid zones between the migratory phenotypes in central Sweden (*N* = 702) and in Poland/Lithuania (*N* = 106) but also included reference samples from each migratory phenotype from southern Scandinavia and Scotland (*N* = 111), Northern Scandinavia, Finland, and Estonia (*N* = 192) (Table S2). Our dataset further included four samples each from Yekaterinburg and Anadyr in Russia. This data allowed us to accurately estimate allele frequencies in allopatric populations and the change in allele frequencies across both of the hybrid zones.

The majority of probes (*N* = 5839) on the SNP array was designed from transcriptome reads (Lundberg et al. 2013) mapped to the zebra finch genome. We used a customized pipeline to extract and filter potential probe sequences surrounding each SNP. The remaining probes were manually designed from restriction‐associated DNA (RAD) data mapped to the zebra finch genome, from Sanger sequences from previous studies (Lundberg et al. 2011; Lundberg et al. 2013), or included as replicates. The raw array data was quality‐trimmed based on minor allele frequency and missingness per locus and genotype. Once the willow warbler genome assembly had been completed, we included only SNPs that had a probe sequence that could be successfully mapped to the assembly. With the filtered genotype data, we explored genetic structure among the sample locations using a PCA‐based clustering with smartpca in the Eigensoft package (Patterson et al. 2006) and by calculating F_ST_ (Weir and Cockerham 1984) in hierfstat (Goudet 2005).

### POPULATION GENOMICS ANALYSES

For the whole‐genome resequencing data we estimated genetic differentiation and diversity within and between migratory phenotypes for each variant (SNPs and indels) and for biallelic SNPs in windows of 10 kb (see Supplementary methods for further details). The window estimates were filtered based on their number of callable sites, that is the number of sites for which a polymorphism could have been detected following variant filtering criteria (e.g., coverage). Genetic differentiation was quantified as F_ST_ (Weir and Cockerham 1984) in vcftools, with a weighted average over 10 kb windows. We further explored which genes and gene functions were associated with variants with F_ST_≥0.7, the top 1% most differentiated windows and array SNPs with a F_ST_≥0.1. Individual variants were annotated by using SNPeff (Cingolani et al. 2012) and the position of 10 kb windows relative to annotated genes was determined using bedtools (Quinlan and Hall 2010).

### GENETIC STRUCTURE WITHIN DIFFERENTIATED REGIONS

We used a multidimensional scaling (MDS)‐based method in the R package invclust (Caceres and Gonzalez 2015) to visualize the genetic structure between southern and northern haplotypes (i.e., haplotypes most common in either of the migratory phenotype) in each differentiated region. With very restricted or absent recombination between southern and northern haplotypes, samples will fall into either of three equidistant clusters, with homozygote carriers of southern (SS) and northern (NN) haplotypes at each end, respectively, and heterozygous carriers (NS) clustering in the middle. This clustering is conceptually similar to the genetic structure of two genetically distinct populations that hybridize but only give rise to form F1 hybrids, which form a distinct equidistant cluster in between the parental populations. By limiting the analysis to particular chromosome regions, this method has been developed to identify inversion polymorphisms, that is regions of reduced recombination between distinct groups of haplotypes, in humans and classify individuals as carriers of collinear or inverted haplotypes. For this analysis, SNP genotypes within each differentiated chromosome region were analyzed jointly to provide coordinates of each sample along the two major axes of variation.

Once samples had been assigned a genotype of southern and northern haplotypes (SS, NS, or NN) for each of the three divergent regions, we investigated the geographical distribution of each haplotype within each chromosome region. Specifically, we fitted geographical clines to the frequency of the northern haplotypes at each sampling site using the R package HZAR (Derryberry et al. 2014). We also examined whether the migratory phenotypes are more strongly associated with variation in any of the three differentiated regions, and more closely associated with these regions than other phenotypic traits (e.g., size measurements) that show some difference between the willow warbler subspecies. For this purpose we calculated the frequency of the northern haplotypes for each geographical sampling site and differentiated chromosome regions, as well as the sampling site mean of the following routinely measured phenotypic traits: the ratio of stable nitrogen isotopes in feathers (proxy for migratory phenotype), wing length, tarsus length, bill‐head length, and a color score (1–9) quantifying the whiteness on the breast relative to three reference specimens (see Bensch et al. 2009 for details). Because of the high correlation between the three length measurements we combined them into a single size measurement using the first principal component (58% of variation) from a PCA. We restricted the analyses to males (*N* = 1029, Table S3) as females differ from males in size measurements. The strength of the relationship between allele frequency and phenotypes was quantified using a Pearson correlation coefficient in R. These analyses are based on mean values per sampling sites rather than on the individual samples because the migratory trait (measured from feather stable isotopes) shows large individual variation within each migratory phenotype (Bensch et al. 2006). However, this approach is accurate enough to assign a population sample to either of the two different African wintering areas, or to a population with either mixed or intermediate migration strategies (Bensch et al. 2009).

### GENETIC VARIATION WITHIN DIFFERENTIATED CHROMOSOME REGIONS

The pattern of genetic variation within each group of northern and southern haplotypes combined with the genetic divergence between them provides additional information about the effect of selection, recombination rate, and time since the haplotype groups separated. For example, reduced variation in these regions compared to the rest of the genome, could be indicative of low recombination rate, which would make the variation‐reducing effect of background selection more pronounced. Similarly, low recombination rate could also lead to reduced variation by increasing the magnitude of selective sweeps arising from positive selection. We performed these analyses by dividing the resequencing data into two sets of individuals that were consistently southern (*N* = 8) or northern homozygous (*N* = 6) for all three differentiated chromosome regions (Table S1). This division was based on the same MDS approach as used above, but restricted to a subset of array SNPs that had been genotyped in the resequencing data. Once the resequenced samples had been assigned to groups of pure northern and southern individuals, we used vcftools to estimate nucleotide diversity and Tajima's D, and customized perl scripts to calculate d_XY_ and d_A_ in windows of 10 kb. We tested for significance between mean genetic variation estimates for each of the chromosome regions and the rest of the genome using an ANOVA and Tukey's HSD test in R (R Development Core Team 2014.) for pairwise significance between estimates.

### BREAKPOINT ANALYSES AND DETECTION OF STRUCTURAL VARIANTS

We used delly version 0.65 (Rausch et al. 2012) with default settings to identify structural variants from discordantly aligned read pairs and split reads. Structural variant calls were further quality filtered using the delly script populationFilter.py.

Breakpoints could potentially be located in nonassembled parts of the genome between the scaffolds at the ends of the regions and adjacent scaffolds. We therefore designed primers that could amplify across gaps between the ends of the regions and their neighboring scaffolds as determined from alignments to other bird genomes. For this purpose, we used a long‐range PCR kit (Qiagen, CA, USA) following instructions from the manufacturer. For the PCR assays we used DNA from two northern samples (01L/19 and 01L/20) and one southern sample (01P/02) that were included in the whole‐genome sequencing (Supplementary methods).

## Results

### GENOME ASSEMBLY

The final assembly comprised 5996 scaffolds with a scaffold N50 of 3.2 Mb and total length of 1.07 Gb. Most of the larger scaffolds (*N* = 956, sum = 1.03 Gb) could at least partially be mapped to the genomes of chicken, zebra finch, and collared flycatcher, and many of them mapped to all three genomes (*N* = 667, sum = 994 Mb). Whole‐genome alignments demonstrated high conservation in chromosomal position between these species and suggested few large‐scale changes in genome structure that were unique to the willow warbler.

### WHOLE‐GENOME RESEQUENCING

**Figure 1:**
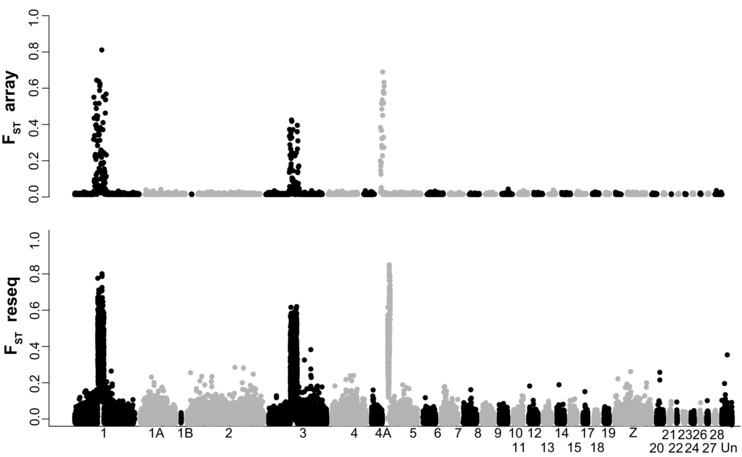
Genetic differentiation (F_ST_) across scaffolds ordered according to alignments to zebra finch chromosomes. Scaffolds have been assigned to a particular chromosome if the mapped interval spans at least 40% of their length and have otherwise been concatenated into an unplaced chromosome (Un). Top: estimates for 4063 SNPs included on the array, calculated between 111 southern and 181 northern individuals. Bottom: weighted average for nonoverlapping 10 kb windows between nine southern and nine northern resequenced individuals.

With ∼9× coverage whole‐genome resequencing data (excluding sequence duplicates) of nine individuals per migratory phenotype mapped to the assembly, we were able to detect 40 million SNPs or indels. The vast majority of variants (35.7 million) were biallelic SNPs, of which 14.2 million (40%) were present as singletons. Overall genetic differentiation between the migratory phenotypes was very low with a total weighted average F_ST_ of 0.007 among biallelic SNPs and a mean F_ST_ among nonoverlapping 10 kb windows of 0.0107 (95 CI: 0.0105–0.0109). Highly differentiated variants were clustered in three regions on chromosomes 1, 3, and 5 (Fig. 1), comprised of two (11.6 Mb), one (13.1 Mb), and at least 10 scaffolds (4.0 Mb), respectively (Fig. S1). Together, these regions comprise approximately 2.7% of the assembled genome. The highly differentiated regions on chromosome 3 and 5 were located close to predicted centromeres based on synteny to the zebra finch and chicken genomes, while on chromosome 1, the centromere was predicted to be ∼40 Mb further downstream of the differentiated region.

Only 33 SNPs and indels with F_ST_≥0.7 were found outside the three differentiated chromosome regions and located on 29 scaffolds (Table S4), compared to 10,934 found in the three regions. The top 1% of the most differentiated filtered 10 kb windows (*N* = 690) were restricted to the differentiated chromosome regions. The most differentiated window outside the differentiated regions (F_ST_ = 0.39) was located in a scaffold that maps further downstream on chromosome 3 in the zebra finch. In the mitochondrial scaffold we identified 223 variable sites, all of which were undifferentiated (F_ST_ = 0) between the migratory phenotypes.

### SNP ARRAY DATA

**Figure 2:**
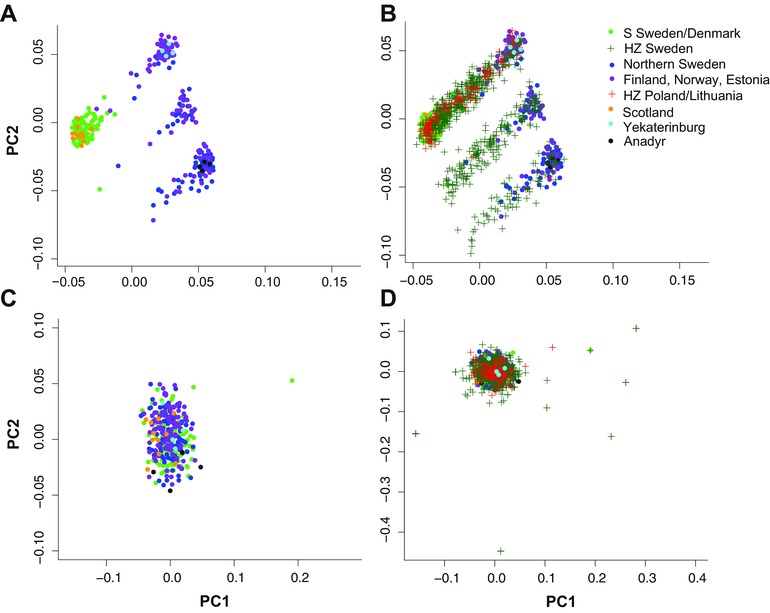
Principal component‐based clustering of genotypes from 4063 SNPs on the array. (A) Only samples outside of the hybrid zones (*N* = 416). (B) All samples (*N* = 1108). (C) Clustering of samples outside of the hybrid zones only including SNPs outside the divergent chromosome regions (*N* = 3777). (D) All samples clustered based on SNPs only outside of the divergent chromosome regions.

We obtained genotypes of 4063 SNPs from 1108 individuals sampled from a total of 74 locations with good representation of both migratory phenotypes and putative hybrids (Table S2). Overall genetic differentiation between the migratory phenotypes was very low, with a weighted average F_ST_ of 0.0157. Highly differentiated SNPs were extremely few (e.g., F_ST_≥0.5, *N* = 24) and localized in the three differentiated chromosome regions identified in the resequencing data (Fig. 1). The highest F_ST_ estimate outside of the differentiated chromosome regions was 0.03. The PCA‐based population clustering separated migratory phenotypes along the first principal component, which was driven by variation in the three differentiated chromosome regions (Fig. 2). The second principal component separated samples within migratory phenotypes based on their genotypes in the differentiated region on chromosome 3, which from the top to bottom represent samples homozygous for the southern haplotype, heterozygotes, and homozygous northern haplotypes, respectively. The SNP array data further show that the clustering of genotypes of willow warblers in Northern Europe and Asia is driven by three highly differentiated chromosome regions (Fig. 2). If these regions are not considered, there is no apparent genetic difference between even the most distantly located sample sites in Britain and Far Eastern Russia (>6000 km apart) (Fig. 2).

### DETECTION OF STRUCTURAL VARIATION

Over the whole genome we identified 4817 deletions (median length: 1018 bp, range: 501–2,111,000 bp), 774 duplications (median length: 1682 bp, range: 505–4,511,000 bp), 449 inversions (median length: 1896 bp. range: 501–5,181,000 bp) and 487 translocations between scaffolds. The most differentiated structural variants (F_ST_≥0.7, *N* = 4) were limited to the differentiated regions on chromosome 1 and 5. However, none of the structural variants coincided with the ends of the differentiated regions. For both the differentiated regions on chromosome 1 and 3, long‐range PCR resulted in amplification across the gap formed by one of the ends and an adjacent scaffold, but failed at the other end. We were only able to get PCR products of the expected gap sizes in the two northern samples, but in this case the amplification had been unspecific and resulted for each locus in multiple bands on an agarose gel. The sequences of these products could not be recovered with Sanger sequencing, and thus did not allow for a localization of the breakpoints.

### GEOGRAPHICAL DISTRIBUTION OF DIFFERENTIATED HAPLOTYPES

**Figure 3:**
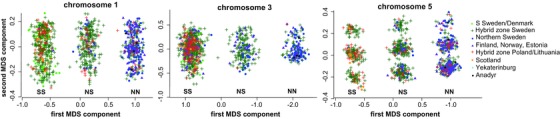
Multidimensional scaling of array genotypes from SNPs located within each of the three divergent chromosome regions. Each genotype cluster is based on the geographical origin of most of its samples and has been assigned as either southern homozygous (SS), northern homozygous (NN), or heterozygous (NS). Symbols referring to the specific locations are listed in the legend, in brief for reference: Sweden/Denmark (green circle), Hybrid Zone Sweden (green plus), Northern Sweden (blue circle), Finland, Norway, and Estonia (purple triangle), Hybrid zone Lithuania/Poland (red plus), Scotland (orange circle), Yekaterinburg (light blue circle), and Anadyr (black circle).

We applied a multidimensional scaling (MDS) approach to the SNP array data to genotype the individuals for southern and northern haplotypes in each of the differentiated chromosome regions. This analysis revealed three distinct and equidistant genotype clusters for each chromosome region, with homozygotes for each haplotype group at the ends, and heterozygotes in between (Fig. 3). The clearly defined groups suggest very restricted recombination between haplotypes of each cluster.

**Figure 4:**
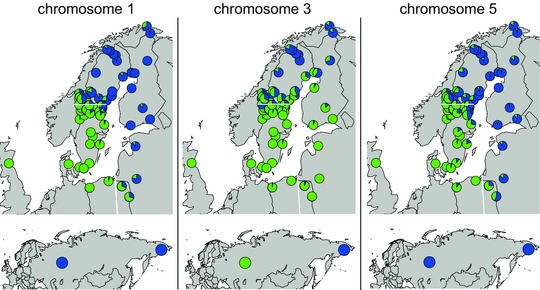
Geographical distribution of southern (green) and northern (blue) haplotypes for each of the three divergent chromosome regions. White lines represent approximate borders between the distributions of the migratory phenotypes. The upper panel represents the North European distribution and the lower panel distribution found at two Russian sites. The breeding distribution of the species covers most of Eurasia north of 40 degree latitude.

Variation in the regions on chromosomes 1 and 5 show the strongest correlation with the migratory phenotypes (measured as the site mean of stable nitrogen isotope ratios in feathers) compared to any of the other measured phenotypic traits that show some difference between the subspecies (Fig. S2, Table S5). Also, these correlations are stronger for the regions on chromosomes 1 and 5 compared to chromosome 3, and remained consistent when analyzed separately for the sampling sites in Sweden and east/south of the Baltic, that is along transects intersecting each of the two migratory divides located in central Scandinavia and eastern Poland, respectively (Fig. 4). In contrast, the geographical distribution of northern alleles in the region on chromosome 3 is more associated with high altitude and latitude environments (Fig. 4).

All three chromosome regions showed narrow (271–688 km) sigmoid frequency clines of southern and northern haplotypes across the hybrid zone in central Sweden (Fig. S3, Table S6) compared to a scenario that would be expected with neutral diffusion given a dispersal distance of 50 km and 100 generations since secondary contact (Bensch et al. 1999). The migratory phenotype‐specific regions on chromosomes 1 and 5, showed a similar pattern in the hybrid zone in Poland and Lithuania with cline widths of 373 and 249 km, respectively. Many individuals in the hybrid zones were heterozygous for both of the regions on chromosome 1 and 5, which suggests that hybridization between migratory phenotypes is frequent. All other possible genotype combinations were also present, which is suggestive of later generation hybrids and backcrosses (Fig. S4).

### GENETIC VARIATION WITHIN DIFFERENTIATED CHROMOSOME REGIONS

**Figure 5:**
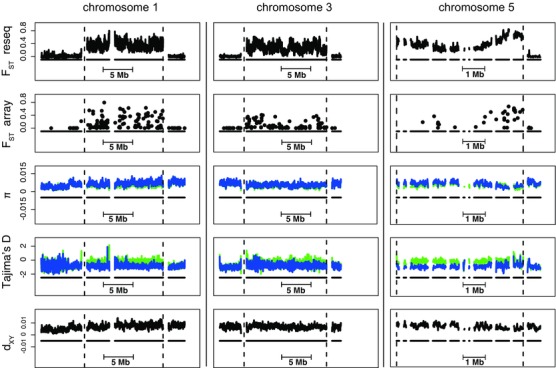
Genetic variation (from the top F_ST_ for resequencing data, F_ST_ for array SNPs, nucleotide diversity (π), Tajima's D, and d_XY_, respectively) within each of the three divergent chromosome region (within dashed lines) and the nearest surrounding scaffolds. For nucleotide diversity and Tajima's D, estimates for southern and northern individuals have been marked with green and blue, respectively. With the exception of F_ST_ for SNPs on the array, estimates have been summarized for nonoverlapping 10 kb windows and been filtered for a minimum number 5000 callable sites, and in the case of F_ST_, also for a minimum of 25 SNPs. The distance in between scaffolds (solid black lines) has been added for illustrative purposes. Due to the uncertainty regarding which scaffold is located upstream of the region, the plot of chromosome 5 starts with the first scaffold in the divergent region.

We characterized the pattern of genetic variation in and between each of the southern and northern haplotype groups for each divergent region to infer the potential effects of recombination and selection in the regions. Here, we used a subset of resequenced individuals that through MDS clustering had been genotyped as either homozygous northern (*N* = 6) or southern (*N* = 8) for all the three regions. Outside of the differentiated chromosome regions, the average nucleotide diversity in 10 kb windows was 0.005 in both migratory phenotypes, which is about twice as high as in other migratory songbirds (Ellegren et al. 2012; Irwin et al. 2016). Within the differentiated regions on chromosomes 1 and 5 the northern migratory phenotype showed slightly higher nucleotide diversity (0.007) than the southern migratory phenotype (0.005) and the genome on average (0.005) (Fig. 5, Fig. S4, Table S5). In the differentiated region on chromosome 3, individuals of both migratory phenotypes showed levels of diversity similar to the rest of the genome. There was a general excess of rare alleles, with an average Tajima's D of –0.87 in both migratory phenotypes. In the differentiated regions, Tajima's D was clearly higher for southern individuals (Fig. 5, Fig. S5, Table S7).

Absolute divergence (d_XY_) between migratory phenotypes was higher on average in the differentiated chromosome regions compared to the rest of the genome, especially prominent in the regions on chromosomes 1 and 5 (Fig. 5, Fig. S5). Similarly, the net number of nucleotide substitutions (d_A_) was higher on average in the differentiated regions compared to the rest of the genome (Fig. S5, Table S7). The highest window‐based estimates (0.005) were obtained for the regions on chromosome 1 and 3 and would, using substitutions rates from other bird genomes, correspond to a rough divergence time between the haplotype groups of 0.75 and 1.6 Myrs, that is long before the last glaciation.

### FUNCTIONAL VARIATION

We were able to annotate 17,294 protein‐coding genes based on homology to proteins and transcripts from other birds, RNAseq data and ab initio predictions. The differentiated regions on chromosome 1, 3, and 5 contained 146, 135, and 53 genes, respectively. The most differentiated windows on chromosome 1 (F_ST_ = 0.80) were located 30–50 kb upstream of the FRAS1‐related extracellular matrix protein 2 (FREM2) gene, on chromosome 3 within the estrogen receptor (ESR1) gene and Ryanodine receptor 2 (RYR2) gene (F_ST_ = 0.62), and on chromosome 5, 23 kb downstream of the olfactomedin‐like 1 (OLFML1) gene (F_ST_ = 0.85) (Table S8). SNPs and indels with F_ST_≥0.7 (*N* = 10,934) were mostly located several thousand bp or more from the closest gene or within introns with unknown functional consequences. A very small subset of the variants (*N* = 30) was predicted to lead to missense mutations or in‐frame deletions in the divergent regions (Table S3). These mutations were associated with 28 different genes, which were mainly found in the region on chromosome 5 and associated with fatty acid synthesis or olfaction.

Among the most differentiated SNPs included on the array (Table S8) chromosome 1 showed a region with considerably higher differentiation (F_ST_ = 0.80) than any other SNP on the array located 32 kb downstream of the Forkhead Box O1 (FOXO1) gene. On chromosome 3 the highest differentiation (F_ST_ = 0.41) was found in an intron of the Regulator of G‐protein signaling 7 (RGS7) gene, and on chromosome 5, the most differentiated SNP was located 22 kb upstream of the olfactomedin‐like 1 (OLFML1) gene (F_ST_ = 0.67).

## Discussion

By using the willow warbler de novo genome assembly, whole‐genome resequencing and the large‐scale genotyping with a SNP array we have obtained a vastly improved resolution of the genomes of the two willow warbler migratory phenotypes. With this data we were able to identify two chromosome regions that are more strongly associated with the migratory phenotypes than other measured traits and one chromosome region that has genetic variation correlated with altitude and latitude. Each of these chromosome regions show a genetic structure that suggest recombination suppression from inversion polymorphisms and contrast with the overall genome that is extremely similar across the full distribution range of the species.

### POPULATION STRUCTURE OF WILLOW WARBLERS

A striking result is the contrasting pattern found between the three highly differentiated chromosome regions and the otherwise virtually undifferentiated genome (Fig. 1). The absence of differentiation for most of the genome is also apparent across the full breeding distribution range of the species. For example, the 3, 777 loci on the SNP array that were from genomic regions outside of the differentiated regions could not distinguish between samples from Scotland and eastern Siberia, even though the populations are located more than 6000 km from each other (Fig. 2). The overall lack of population structure is also reflected in the mitochondrial genome, which typically differentiates faster between geographically isolated populations than nuclear loci (Avise et al. 1987; Zink and Barrowclough 2008). Despite being able to assess genetic variation for the entire mitochondrial genome, we were not able to detect any sequence differences between the migratory phenotypes. This absence of population structure in most of the genome is likely the result of a recent range expansion involving large effective population sizes and facilitated by high gene flow among expanding populations, although their relative effects remain to be investigated. However, the overall homogenous pattern strongly contrasts with the distinct geographic distributions of the haplotypes at the three differentiated chromosome regions, patterns that are unlikely to be maintained unless selection is operating. The high divergence times separating the haplotype groups (estimated to be 0.75–1.6 million years) suggest that they must have existed together during several glacial episodes and raises further questions about their origin.

In contrast to the willow warbler, two differentially migrating subspecies of Swainson's Thrush *Catharus ustulatus* and two species of *Ficedula* flycatchers show much more elevated levels of genomic background differentiation and a larger number of highly differentiated regions (Ellegren et al. 2012; Ruegg et al. 2014). The lack of differentiation over most of the genome suggests that the willow warbler migratory phenotypes are at a very early stage of divergence, which is reminiscent of the pattern seen between hooded crows *Corvus cornix* and carrion crows *Corvus corone* where low genome‐wide differentiation contrasts with a 2 Mb highly differentiated region on chromosome 18 (Poelstra et al. 2014). Among migratory birds, differentially migrating barn swallow *Hirundo rustica* populations in Europe show comparable levels of genome‐wide differentiation to the willow warblers (von Rönn et al. 2016). Further worthy of note is that the Z chromosome, which is believed to play an important role in reproductive isolation among birds (Price and Bouvier 2002; Qvarnström and Bailey 2009), did not show any evidence of increased differentiation between the migratory phenotypes. Finally, the early stage of divergence between the migratory phenotypes is further supported by a lack of assortative mating (Liedvogel et al. 2014) and the extensive mixing in the hybrid zones including putative backcrosses and later generation hybrids (Fig. S4).

### ASSOCIATION BETWEEN GENETIC VARIATION AND MIGRATORY PHENOTYPES

We did not find any differences related to previously suggested candidate genes or chromosome regions for migration, such as ADCYAP1 in blackcaps (Mueller et al. 2011) or a large region (∼30 Mb) on chromosome 4 identified in the Swainson's Thrush (Delmore et al. 2016). With the new genomic dataset we were able to precisely delineate the three regions located on chromosomes 1, 3, and 5 that in previous studies had been shown to contain loci with exceptionally high differentiation (Lundberg et al. 2011; Lundberg et al. 2013). Each differentiated region spans 4.0 to 13.1 Mb and contains between 53 and 146 protein‐coding genes. Genotypes from a large number of samples around the Baltic Sea suggest that the spatial distribution of alleles within the regions on chromosome 1 and 5 mirror the geographical breeding distribution of the migratory phenotypes (Fig. 4). This relationship is further strengthened by correlations between allele frequency and mean phenotypic measurements at each site (Fig. S2, Table S5). Both chromosome regions show the strongest correlation with stable nitrogen isotope ratios in feathers, which is a proxy for wintering area. Importantly, this strong association with migratory phenotype is also observed when restricting the analysis to sites on the eastern/southern side of the Baltic Sea, which is previously known to differ more in cline shapes between the phenotypic traits than on the western side (Bensch et al. 2009). The region on chromosome 3 also shows high differentiation between the migratory phenotypes across the migratory divide in Scandinavia Sweden, albeit lower than in the other two regions. However, it is not correlated to the migratory phenotypes across the migratory divide located on the eastern/southern side of the Baltic. Instead, the variation within this chromosome region shows the highest correlation with altitude and latitude (Fig. 4). This environmental correlation was previously demonstrated for a single biallelic marker located within this chromosome region (Larson et al. 2014).

### GENETIC STRUCTURE WITHIN DIFFERENTIATED REGIONS

The large size of the differentiated regions, each comprising several Mb and containing many coding genes, is intriguing. “Genomic islands of differentiation” have been attributed to processes such as background or positive selection in regions of low recombination, for example close to centromeres (Cruickshank and Hahn 2014), which has will lead to decreased within‐population diversity and possibly to increased relative differentiation (e.g., F_ST_), but not increased absolute divergence (d_XY_). These processes have been associated with the formation of large differentiated regions in several lineages of birds (Burri et al. 2015; Irwin et al. 2016). Indeed, two of the regions (on chromosome 3 and 5) correspond to regions of the zebra finch genome located close to, or overlapping with centromeres (Fig. S1). However, we did not observe any clear reduction in nucleotide diversity or excess of rare alleles within the divergent regions in either migratory phenotype, suggesting that the recombination rate (within the migratory phenotypes) is not reduced in these regions. All three regions showed elevated mean absolute divergence (d_XY_) between migratory phenotypes compared the average of the genome, and was most pronounced for the regions on chromosomes 1 and 5. Taken together, these patterns suggest that it is unlikely that low recombination rate combined with background or positive selection is the main driving process behind the differentiated regions. The strong “block‐like” inheritance of southern and northern alleles rather suggests the presence of inversion polymorphisms, which would result in limited recombination between inverted and noninverted haplotypes, but not within each group of haplotypes.

The lack of recombination between southern and northern haplotypes is strongly supported by the well‐defined and equidistant genotype clusters (Fig. 3). Similar patterns have been observed in some experimentally validated inversions in human (Caceres and Gonzalez 2015) as well as in PCA‐based clustering of SNP genotypes in an experimentally validated inversion on chromosome 5 in the zebra finch genome (Knief et al. 2016). We were not able to confirm or refute the presence of breakpoints through our analyses of discordantly mapped reads and long‐range PCR across scaffolds. However, since many inversion breakpoints are located within repetitive regions, the applicability of these methods is limited (Lucas Lledo and Caceres 2013).

Inversions have recently received attention in evolutionary biology due to their potential for maintaining adaptations despite ongoing gene flow (Hoffmann and Rieseberg 2008). If gene flow is present between two populations that are locally adapted, and certain combinations of alleles at some linked loci are favored in each of them, an inversion spanning these loci would suppress recombination and ensure that the favorable alleles are inherited together, which consequently would drive the inversion to high frequency (Kirkpatrick and Barton 2006). Interestingly, inversion polymorphisms have been associated with migratory phenotypes in cod *Gadus morhua* (Kirubakaran et al. 2016) and mating strategies in ruff *Philomachus pugnax* (Lamichhaney et al. 2016) and white‐throated sparrows *Zonotrichia albicollis* (Thomas et al. 2008). Hence, it is tempting to speculate that several of the ∼200 genes located within the putative inversions on chromosomes 1 and 5 could be associated with the correlated suite of behavioral and physiological traits that are observed in migratory birds (Piersma et al. 2005) and facilitate their coordinated evolution to different migratory routes and wintering areas. The association between variation in the differentiated region on chromosome 3 and high altitude and latitude is also highly interesting as inversion polymorphisms have been associated with environmental gradients in insects (Kapun et al. 2016; Cheng et al. 2012). This is to our knowledge the first time a putative inversion has been associated with an environmental gradient in a bird population.

### FUNCTIONAL VARIATION WITHIN DIFFERENTIATED REGIONS

Due to the high linkage disequilibrium found within the differentiated regions, it is difficult to pinpoint specific targets of selection. Nonetheless, in the case of inversions, limited recombination between haplotypes from each cluster may over time homogenize parts of the regions, leaving the locally adapted genes differentiated (Guerrero et al. 2012). To identify potential candidate genes under selection in each region, we explored the position of the most differentiated windows in relation to annotated genes and highly differentiated nonsynonymous mutations. On chromosome 5, one of the most differentiated 10 kb windows is overlapping the two fatty acid desaturase genes FADS1 and FADS2 (Table S8) involved in biosynthesis of essential fatty acids that have been associated with putative dietary adaptations in humans (Ameur et al. 2012). In addition, several of the genes associated with the small number of highly differentiated missense mutations were involved in fatty acid synthesis or carbohydrate and fatty acid metabolic processes (Table S4). The most differentiated array SNP on chromosome 1, which is noticeably higher than any other in the genome, is located 32 kb downstream of the Forkhead Box O1 (FOXO1) gene (Table S8). This gene encodes a transcription factor that has been demonstrated to play an important role in gluconeogenesis and adipogenesis in mice (Gross et al. 2008). Long‐distance migratory birds primarily use fat as energy and the composition of fatty acids has been associated with flight performance (Price 2010). The migratory phenotypes of the willow warbler differ significantly in the distance they migrate and it is tempting to speculate that these differences represent adaptations in fueling to their different routes.

The most differentiated 10 kb windows and array SNPs on chromosome 3 are located within the RYR2 gene. This gene encodes calcium channels that are involved in heart muscle contraction and has been shown to be under selection in high altitude vertebrates (Zhang et al. 2014; Wang et al. 2015). The authors explained this as an adaptation to hypoxia, which should not be a selective force in willow warblers as the breeding altitudes are typically below 1000 meters, but it is possible that changes within this gene reflect adaptations to increased metabolic demands necessary to cope with the colder climate at higher altitudes and latitudes. One of the two highly differentiated missense mutations in this chromosome region is located within mitochondrial fission regulator 2 (MTFR2) gene, which is involved in aerobic respiration (Table S4).

### CONCLUSION AND FUTURE DIRECTIONS

Our study provides important and previously inaccessible genomic insights into how local adaptations could form and persist between recently diverged and hybridizing taxa, and particularly in the context of migratory divides. To determine whether high differentiation in the three chromosome regions is maintained by inversion polymorphisms will require further evidence, and promising approaches include long read sequencing technologies and optical maps. It is difficult to pinpoint specific targets of selection due to the high linkage disequilibrium in the regions. Nonetheless, the most differentiated intervals and missense mutations within the differentiated regions provide candidate genes involved in migration physiology and cold tolerance in birds, which are poorly understood from a genetic perspective. Future studies on the genomes of other birds showing similar adaptations, but that do not possess the same large differentiated regions, will be useful to further highlight particular genes and pathways that could have been involved in shaping the differentiated regions in the willow warbler.

Editor in Chief: J. Slate

## Supporting information


**Fig. S1**. Order and orientation of scaffolds included in each of the three differentiated regions
based on their synteny to the zebra finch, collared flycatcher and chicken genome.
**Fig. S2**. Correlation between the frequency of northern haplotype in each of the three differentiated chromosome regions and the mean of phenotypic traits (nitrogen isotope ratios, size and color) per sampling site.
**Fig. S3**. Geographic clines for the northern haplotype frequency across the migratory divides in central Sweden (differentiated regions of chromosomes 1, 3 and 5) and Poland/Lithuania (chromosomes 1 and 5).
**Fig. S4**. Frequency distribution of the combined genotypes at the differentiated regions of chromosome 1 ‐ chromosome 5 in allopatric populations of the migratory phenotypes (A) and in the two hybrid zones (B).
**Fig. S5**. Genetic variation within each of the three differentiated chromosome regions compared to the rest of the genome.
**Table S1**. Samples used for whole‐genome resequencing and RAD sequencing, respectively. Average coverage refers to coverage at positions in the genome that is covered by at least one read from the sample and has been calculated following removal of sequence duplicates.
**Table S2**. Sampling sites of willow warblers used for the SNP array. All samples were collected from birds caught on breeding territories.
**Table S3**. Frequency of northern haplotype 76 for each differentiated chromosome region and the mean of phenotypic measurements per sampling site.
**Table S4**. Highly differentiated missense mutations and inframe deletions detected in the three divergent chromosome region and all highly differentiated SNPs and indels located on scaffolds outside of the divergent regions.
**Table S5**. Results of correlation analyses between mean phenotypic trait estimates and differentiated chromosome region northern haplotype frequency per sampling site.
**Table S6**. Estimated parameters for geographical clines of northern haplotypes in each of the three differentiated chromosome regions.
**Table S7**. Statistical significance from testing differences in the mean 10 kb window estimates of genetic variation (nucleotide diversity, Tajima's D, dXY and dA) among each differentiated chromosome region and the rest of the genome.
**Table S8**. Annotation of the 10 most differentiated filtered 10kb windows and array SNPs in each of the divergent chromosome regions. Up‐ and downstream refer to the orientation relative to the transcription of the gene.
**Table S9**. Primers used for long‐range PCR across the ends of the differentiated regions on chromosome 1 and 3 and their adjacent scaffolds determined from whole‐genome alignments to other bird genomes (Fig. S1).
