# Supplementary material for: Genetic differences between willow warbler migratory phenotypes are few and cluster in large haplotype blocks
Source: Evol Lett. 2017 Jun 16;1(3):155–68. doi: 10.1002/evl3.15 (PMC6121796; doi:10.1002/evl3.15)
Supplement: Supplementary file 1 — Fig. S1. Order and orientation of scaffolds included in each of the three differentiated regions based on their synteny to the zebra finch, collared flycatcher and chicken genome. Fig. S2. Correlation between the frequency of northern haplotype in each of the three differentiated chromosome regions and the mean of phenotypic traits (nitrogen isotope ratios, size and color) per sampling site. Fig. S3. Geographic clines for the northern haplotype frequency across the migratory divides in central Sweden (differentiated regions of chromosomes 1, 3 and 5) and Poland/Lithuania (chromosomes 1 and 5). Fig. S4. Frequency distribution of the combined genotypes at the differentiated regions of chromosome 1 ‐ chromosome 5 in allopatric populations of the migratory phenotypes (A) and in the two hybrid zones (B). Fig. S5. Genetic variation within each of the three differentiated chromosome regions compared to the rest of the genome. Table S1. Samples used for whole‐genome resequencing and RAD sequencing, respectively. Average coverage refers to coverage at positions in the genome that is covered by at least one read from the sample and has been calculated following removal of sequence duplicates. Table S2. Sampling sites of willow warblers used for the SNP array. All samples were collected from birds caught on breeding territories. Table S3. Frequency of northern haplotype 76 for each differentiated chromosome region and the mean of phenotypic measurements per sampling site. Table S4. Highly differentiated missense mutations and inframe deletions detected in the three divergent chromosome region and all highly differentiated SNPs and indels located on scaffolds outside of the divergent regions. Table S5. Results of correlation analyses between mean phenotypic trait estimates and differentiated chromosome region northern haplotype frequency per sampling site. Table S6. Estimated parameters for geographical clines of northern haplotypes in each of the three differentiated chromosom [file EVL3-1-155-s001.pdf]

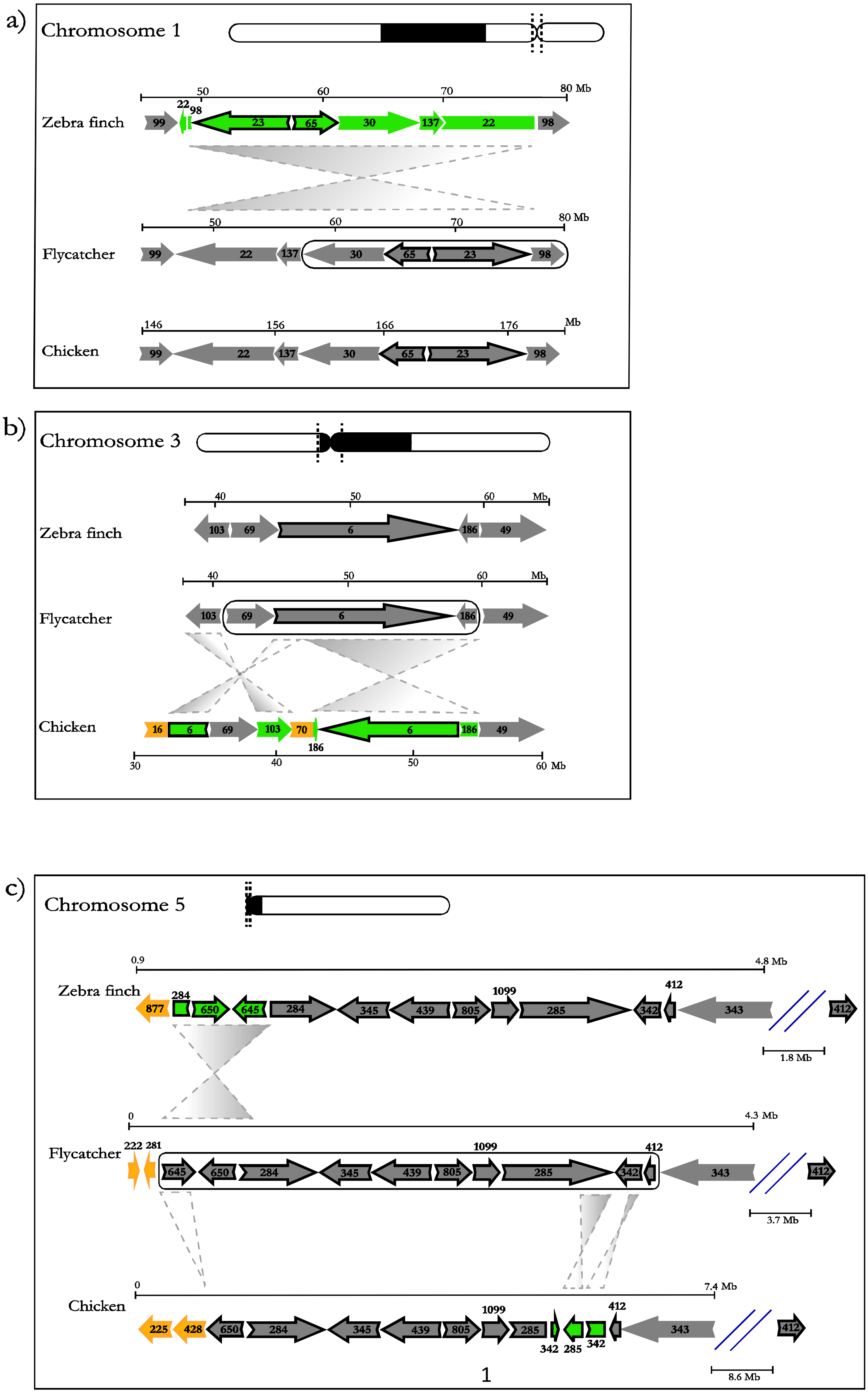
a)
Chromosome 1
Zebra finch
50
60
70
80 Mb
99
22
98
23
65
30
137
22
98
Flycatcher
50
60
70
80 Mb
99
22
137
30
65
23
98
Chicken
146
156
166
176
Mb
99
22
137
30
65
23
98
b)
Chromosome 3
Zebra finch
40
50
60
Mb
103
69
6
186
49
Flycatcher
40
50
60
Mb
103
69
6
186
49
Chicken
16
6
69
103
70
6
186
49
186
30
40
50
60 Mb
c)
Chromosome 5
Zebra finch
0.9
4.8 Mb
877
284
650
645
284
345
439
805
1099
285
342
412
343
412
1.8 Mb
Flycatcher
0
4.3 Mb
222
281
645
650
284
345
439
805
1099
285
342
412
343
412
3.7 Mb
Chicken
0
7.4 Mb
225
428
650
284
345
439
805
1099
285
342
285
342
412
343
412
8.6 Mb
1

Fig. S1. Order and orientation of scaffolds included in each of the three differentiated regions based on their synteny to the zebra finch, collared flycatcher and chicken genome. The black region on top of each figure shows the approximate location of the visualized genomic interval on the corresponding chromosome in the zebra finch genome. The probable location of the centromere of each chromosome is within dashed lines. Green is used to highlight scaffolds that differ in orientation and location relative to the other genomes. Yellow represents scaffolds that are only found in the corresponding regions in one of the genomes. The encircled gene order in each region has been considered the most representative for the willow warbler and been used to visualize genetic variation. One exception is on chromosome 5, where the entire scaffold_412, despite mapping to different locations in the other three genomes, is assumed to be positioned at the end of the divergent region.

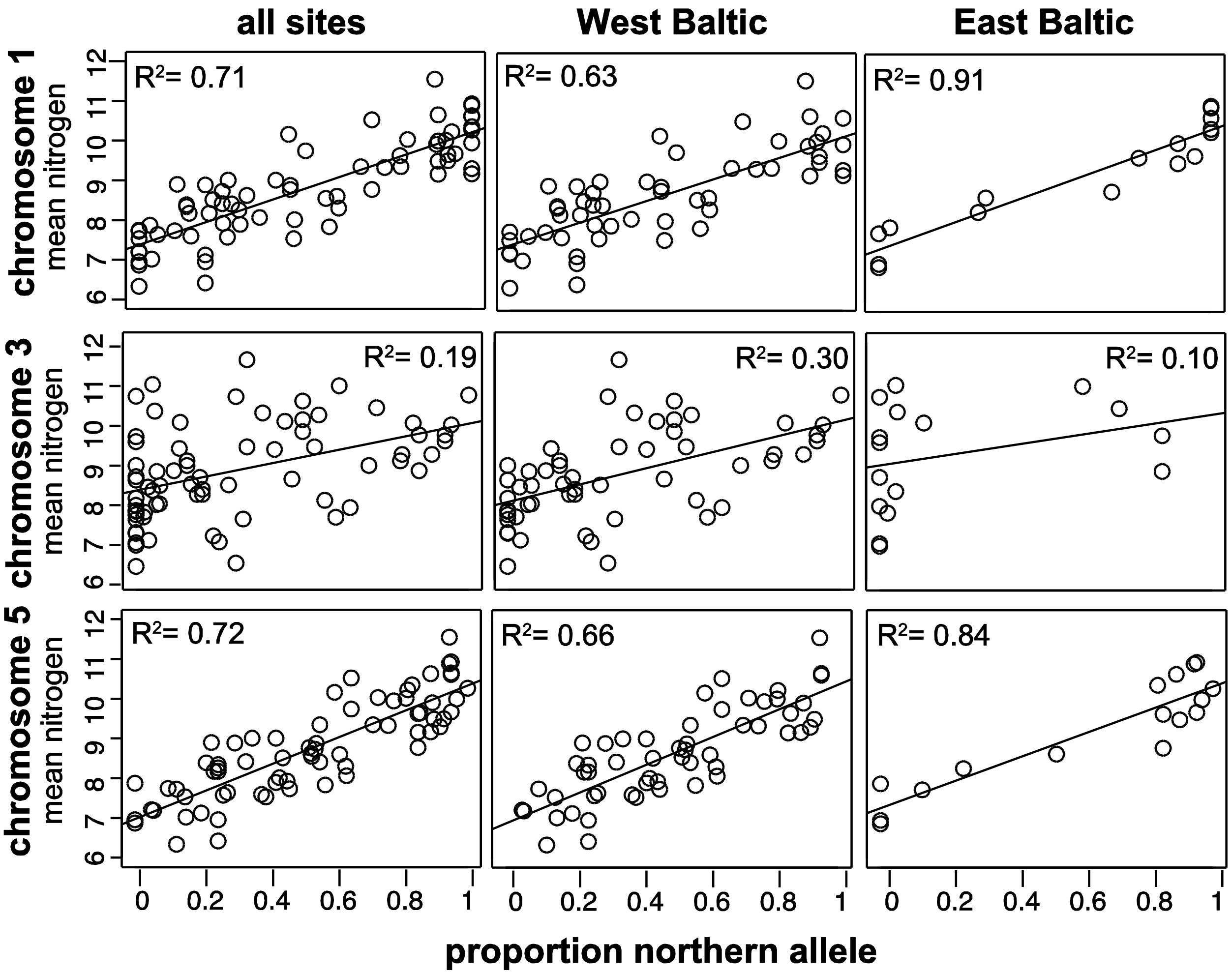
all sites
West Baltic
East Baltic
chromosome 1
chromosome 3
chromosome 5
mean nitrogen
R²= 0.71
R²= 0.63
R²= 0.91
R²= 0.19
R²= 0.30
R²= 0.10
R²= 0.72
R²= 0.66
R²= 0.84
6 7 8 9 10 11 12
0 0.2 0.4 0.6 0.8 1
proportion northern allele

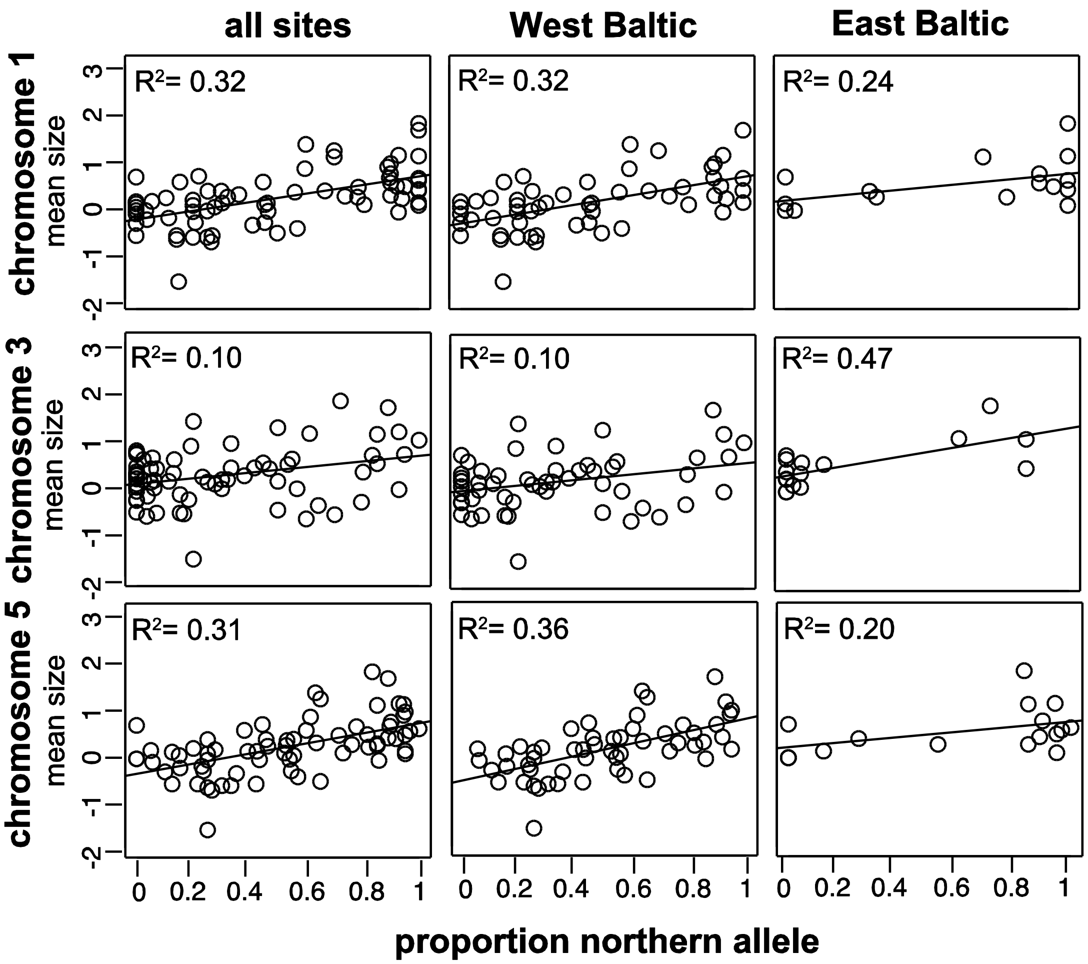
all sites
West Baltic
East Baltic
chromosome 1
chromosome 3
chromosome 5
mean size
R²= 0.32
R²= 0.32
R²= 0.24
R²= 0.10
R²= 0.10
R²= 0.47
R²= 0.31
R²= 0.36
R²= 0.20
proportion northern allele

27

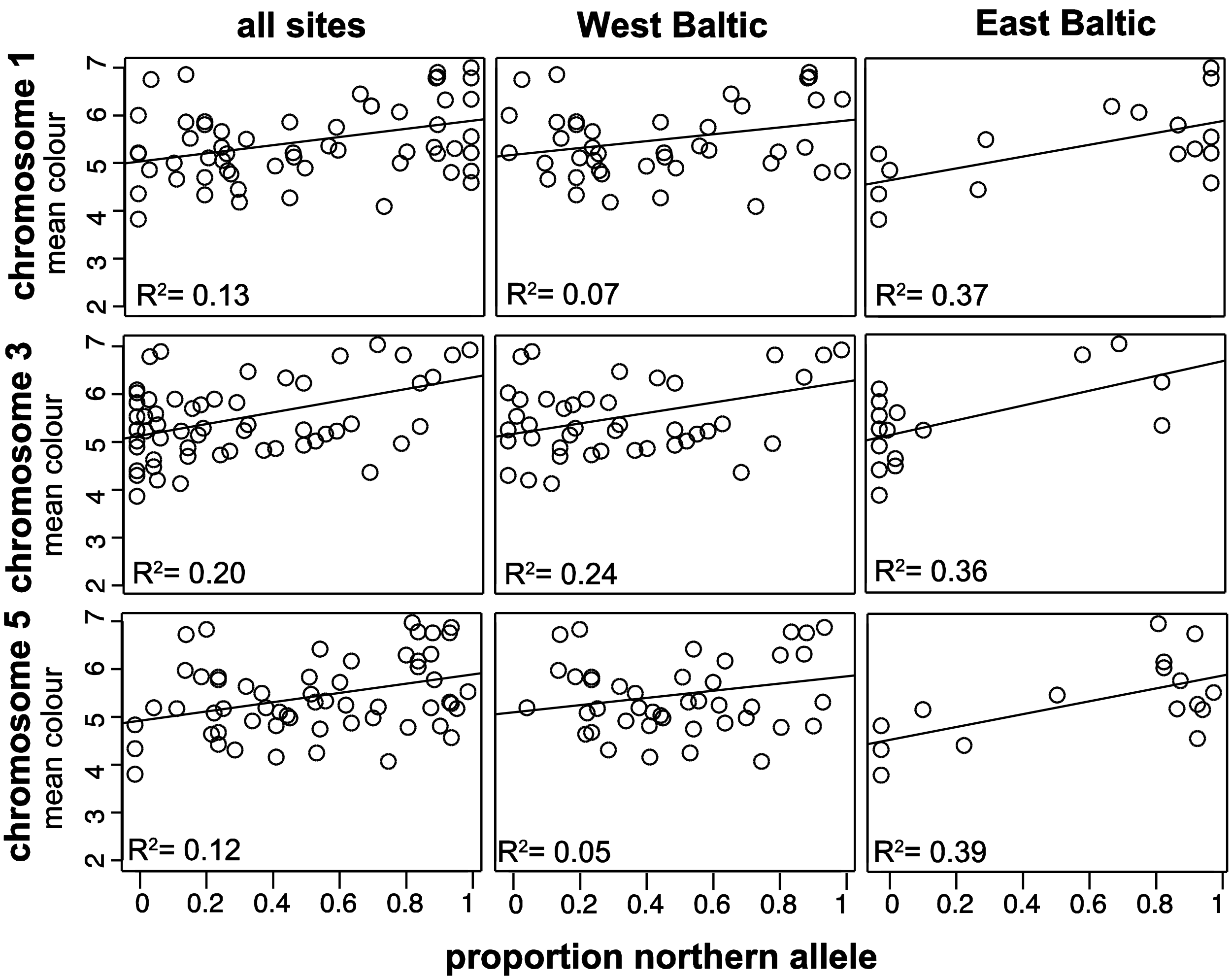

Fig. S2. Correlation between the frequency of northern haplotype in each of the three differentiated chromosome regions and the mean of phenotypic traits (nitrogen isotope ratios, size and color) per sampling site. Each analysis has been performed on males (N=1,029) across all sites and separately for sampling sites in the Western Baltic (Sweden) and in the Eastern/Southern Baltic (Table S3). Size is the first principal component from a PCA on wing length, tarsus length and bill-head length. Color represents a scale between 0-9 that quantifies the amount of whiteness based on a comparison to three reference specimens.

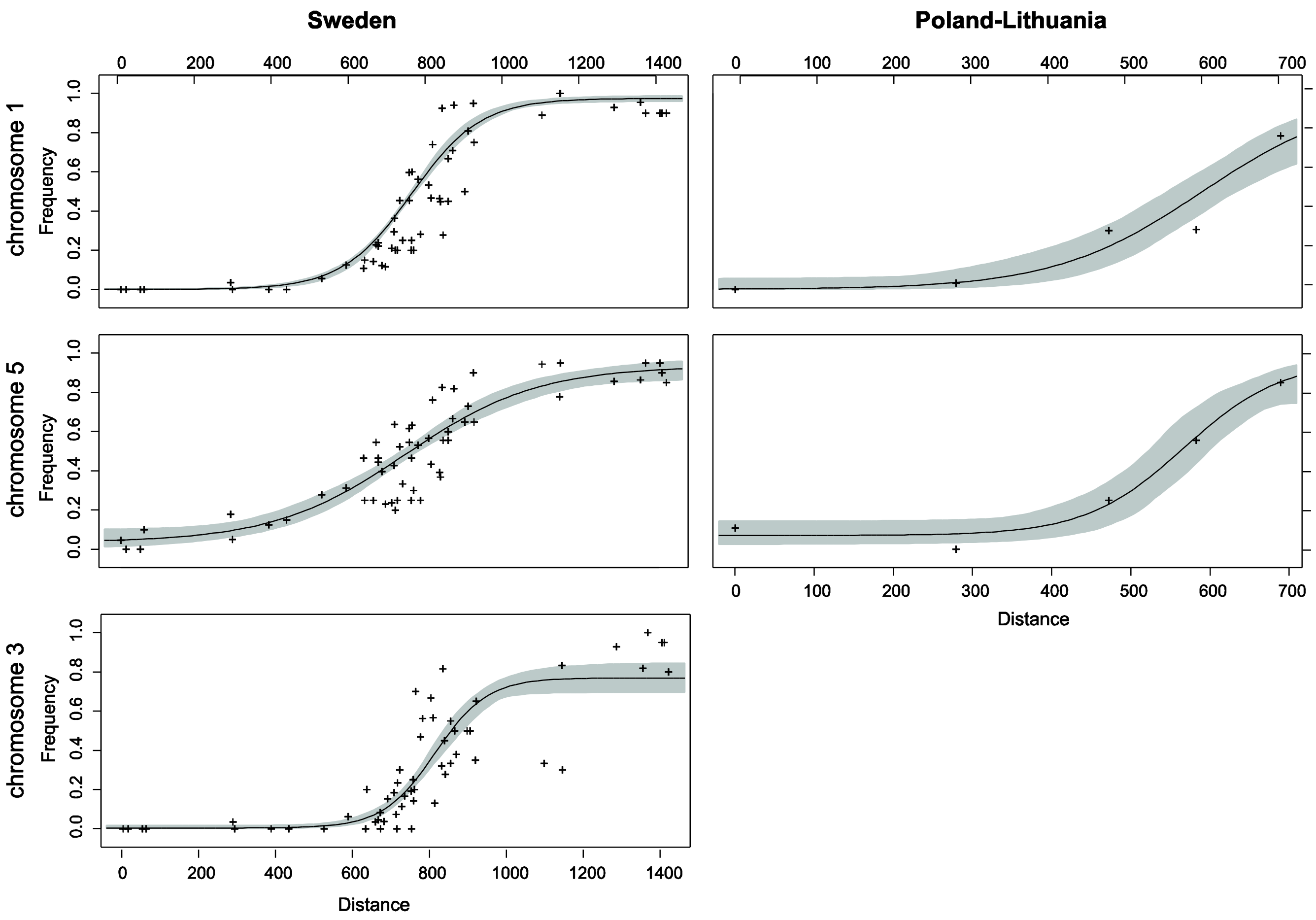

Fig. S3. Geographic clines for the northern haplotype frequency across the migratory divides in central Sweden (differentiated regions of chromosomes 1, 3 and 5) and Poland/Lithuania (chromosomes 1 and 5). Plus symbols represent the observed haplotype frequencies at each site and the shaded area a 95 % confidence interval of the fitted curve.

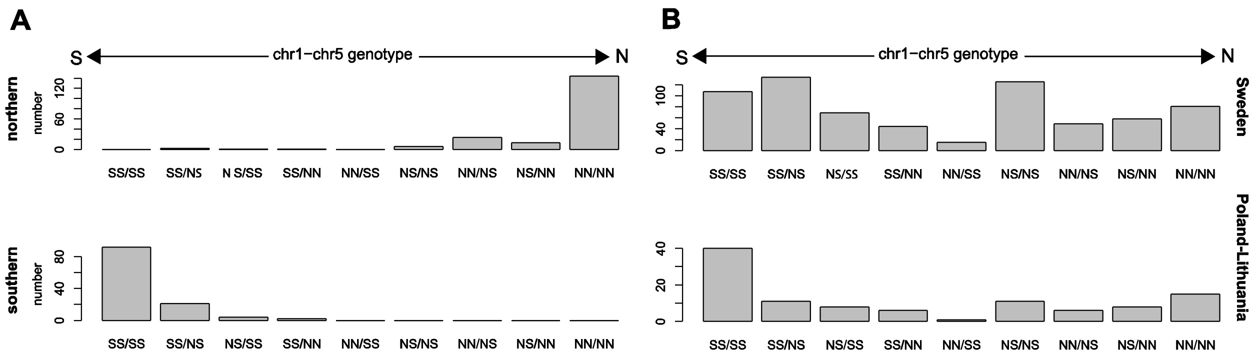

Fig. S4. Frequency distribution of the combined genotypes at the differentiated regions of chromosome 1 - chromosome 5 in allopatric populations of the migratory phenotypes (A) and in the two hybrid zones (B). Southern and northern haplotypes for each locus is denoted with S and N, respectively. Haplotypes have been inferred from a MDS-clustering of SNP genotypes within each chromosome region.

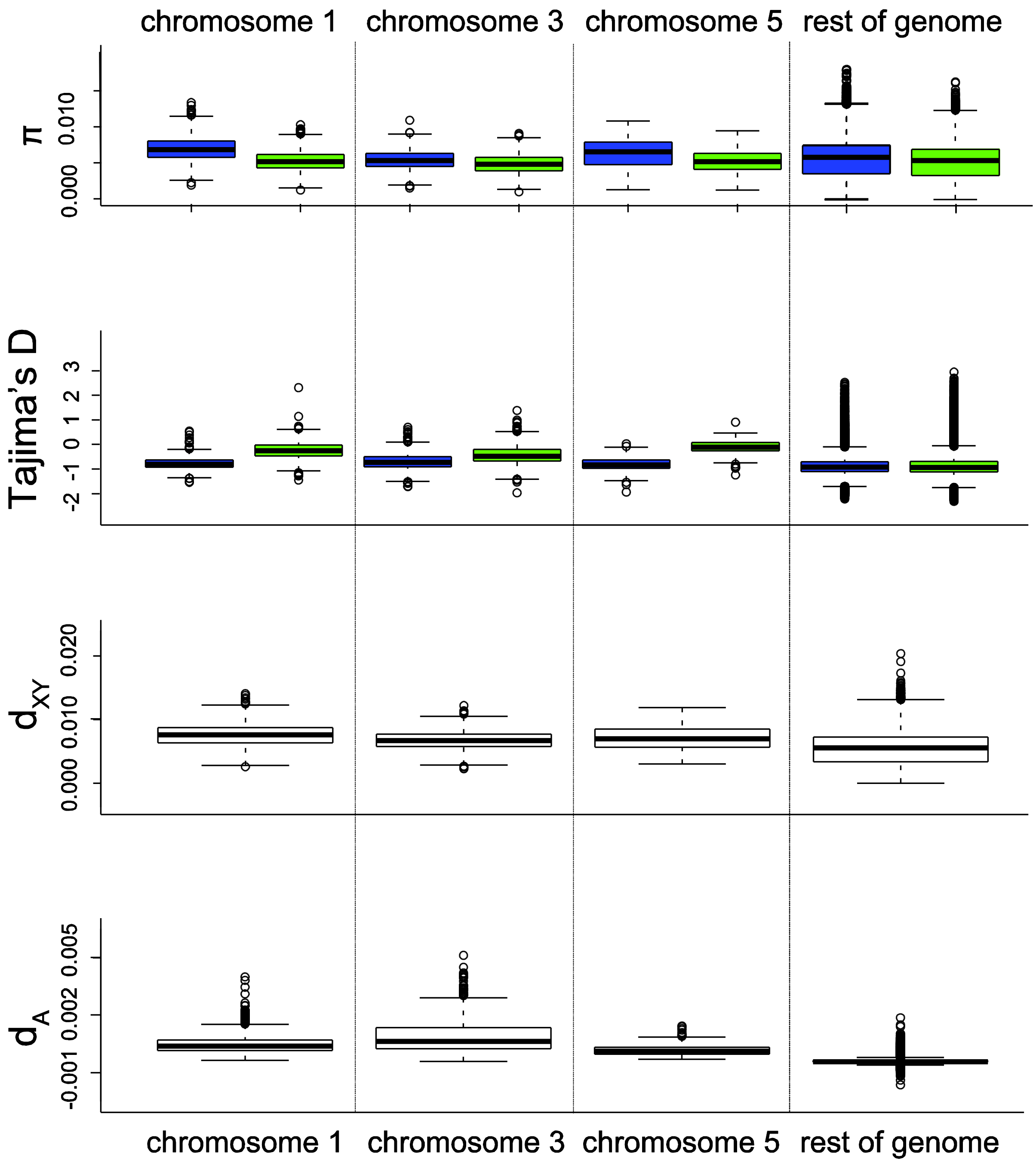

Fig. S5. Genetic variation within each of the three differentiated chromosome regions compared to the rest of the genome. The data is based on 8 samples that were scored as homozygous for southern haplotypes and 6 samples homozygous for northern haplotypes in all the three

differentiated regions. Green and blue boxplots represent estimates for the southern and northern homozygotes, respectively.

Table S1. Samples used for whole-genome resequencing and RAD sequencing, respectively. Average coverage refers to coverage at positions in the genome that is covered by at least one read from the sample and has been calculated following removal of sequence duplicates. Sample 1L19, which was the same as used in the de novo assembly, had very few reads in the resequencing data. In this case we used the 650 bp library from the *de novo* assembly, which gave it a much higher coverage. Samples that were either homozygous southern (N=8) or northern (N=6) for all of the three differentiated chromosome regions are marked with *. For RAD sequencing, reads have been mapped to the zebra finch genome.

Sample	Migratory phenotype/ region	Location	Raw sequences (millions)	Filtered and mapped sequences (millions)	Average coverage
**Resequencing**					
96A/01*	*southern*	Stensoffa, Sweden, 55.7°N, 13.4°E	89	78	7
96B/07*	*southern*	Kvismaren, Sweden, 59.2°N, 15.4°E	118	103	9.2
97A/12*	*southern*	Haganäs, Sweden, 56.3°N, 14.7°E	106	91	8.1
00G/03*	*southern*	Stensoffa, Sweden, 55.7°N, 13.4°E	93	81	7.3
00G/04*	*southern*	Stensoffa, Sweden, 55.7°N, 13.4°E	77	67	6
00G/10*	*southern*	Stensoffa, Sweden, 55.7°N, 13.4°E	98	85	7.6
00J/01*	*southern*	Smedstorp, Sweden, 59.6°N, 15.0°E	98	85	7.6
01P/02*	*southern*	Barkö, Sweden, 60.2°N, 18.2°E	72	114	10.2
03K/06	*southern*	Gräsmarö, Sweden, 58.3°N,17.0°E	110	95	8.5
01K/05	*northern*	Tornehamn, Sweden, 68.4°N, 18.6°E	105	92	8.2
01K/10*	*northern*	Tornehamn, Sweden, 68.4°N, 18.6°E	105	91	8.1
01L/17*	*northern*	Stordalen, Sweden, 68.3°N, 19.1°E	117	102	9.1
01L/19*	*northern*	Stordalen, Sweden, 68.3°N, 19.1°E	538	446	49.1
01L/20*	*northern*	Stordalen, Sweden, 68.3°N, 19.1°E	105	90	8
01M/08*	*northern*	Kaisepakte, Sweden, 68.3°N, 19.3°E	94	82	7.3
01O/01*	*northern*	Kallax, Sweden, 65.5°N, 22.1°E	197	175	15.6

01O/04	*northern*	Kallax, Sweden, 65.5°N, 22.1°E	203	179	15.9
01O/06	*northern*	Kallax, Sweden, 65.5°N, 22.1°E	160	140	12.5
**RAD sequencing**					
96A/01	*Southern*	Stensoffa, Sweden, 55.7°N, 13.4°E	11	4	N/A
96B/07	*Southern*	Kvismaren, Sweden, 59.2°N, 15.4°E	8	3	N/A
03K/06	*Southern*	Gräsmarö, Sweden, 58.3°N, 17.0°E	7	2	N/A
04C/04	*Hybrid zone*	Bialowieza, Poland, 52.7°N, 17.0°E	7	3	N/A
04F/03	*Hybrid zone*	Goleniow, Poland, 53.6°N, 14.7°E	10	3	N/A
00G/03	*Southern*	Stensoffa, Sweden, 55.7°N, 13.4°E	9	3	N/A
00G/04	*Southern*	Stensoffa, Sweden, 55.7°N, 13.4°E	7	2	N/A
00G/10	*Southern*	Stensoffa, Sweden, 55.7°N, 13.4°E	6	2	N/A
04E/02	*Hybrid zone*	Gdansk, Poland, 54.3°N, 18.9°E	12	4	N/A
97A/12	*Southern*	Haganäs, Sweden, 56.3°N, 14.7°E	8	3	N/A
96M/05	*Northern*	Ammarnäs, Sweden, 66.0°N, 16.1°E	7	2	N/A
01K/05	*Northern*	Tornehamn, Sweden, 68.4°N, 18.6°E	8	3	N/A
01K/10	*Northern*	Tornehamn, Sweden, 68.4°N, 18.6°E	5	2	N/A
01L/17	*Northern*	Stordalen, Sweden, 68.3°N, 19.1°E	7	2	N/A
01L/19	*Northern*	Stordalen, Sweden, 68.3°N, 19.1°E	8	3	N/A
01L/20	*Northern*	Stordalen, Sweden, 68.3°N, 19.1°E	12	4	N/A
01M/08	*Northern*	Kaisepakte, Sweden, 68.3°N, 19.3°E	6	2	N/A
01O/01	*Northern*	Kallax, Sweden, 65.5°N, 22.1°E	11	4	N/A
01O/04	*Northern*	Kallax, Sweden, 65.5°N, 22.1°E	7	3	N/A
01O/06	*Northern*	Kallax, Sweden, 65.5°N, 22.1°E	6	2	N/A

Table S2. Sampling sites of willow warblers used for the SNP array. All samples were collected from birds caught on breeding territories. The total number of samples refers to the number of unique samples hybridized to the array and the trimmed number to samples remaining after quality trimming.

Site	Country	Lat	Long	Years	N total	N trimmed
Southern migratory phenotype					111	111
Fife	United kingdom	56.2	3.1	N/A	11	11
Stensoffa	Sweden	55.7	13.4	2001, 2002	11	11

Gadevang Mose	Denmark	55.8	12.5	2005	11	11
Kasted Mose	Denmark	56.2	10.1	2005	12	12
Haganäs	Sweden	56.3	14.7	1997	15	15
Gräsmarö	Sweden	58.3	17.0	2001, 2003	14	14
Tåkern	Sweden	58.3	14.8	1997	10	10
Kvismaren	Sweden	59.2	15.4	1996	8	8
Smedstorp	Sweden	59.6	15.0	2003	10	10
Orböle	Sweden	60.4	18.0	1996	9	9
Swedish hybrid zone					702	692
Aspberget	Sweden	61.0	12.5	2002	8	8
Varbergsviken	Sweden	61.4	17.1	2000	14	14
Bunkris	Sweden	61.4	13.6	1996	10	10
Västbacka	Sweden	61.6	14.8	2010	15	14
Klångstavallen	Sweden	61.7	16.8	2001	33	33
Mörtsjön	Sweden	61.7	17.4	2000, 2001, 2002, 2010	85	84
Hudiksvall	Sweden	61.7	17.4	1996	9	9
Tanna	Sweden	61.8	16.9	2000, 2001	54	53
Idre	Sweden	61.9	12.7	1996, 2010	13	13
Uftberget	Sweden	62.0	14.1	1997, 2010	20	19
Bäcksand	Sweden	62.1	17.4	2000, 2001, 2010	35	34
Gnarp	Sweden	62.1	17.5	1997	11	11
Lofsdalen	Sweden	62.1	13.4	2001	15	15
Sörvattnet	Sweden	62.2	13.0	2001	10	10
Björkön	Sweden	62.2	17.6	2001	23	22
Gimsjön_Ulvsjön	Sweden	62.3	16.5	2001	6	6
Sundsvall	Sweden	62.4	17.4	1996, 2000	26	26
Alnö	Sweden	62.4	17.4	2010	11	11
Vemdalsskalet	Sweden	62.5	14.7	2002	10	10
Rätan	Sweden	62.5	14.6	2010	16	14
Midlanda	Sweden	62.5	17.4	2001	15	15
Tänndalen	Sweden	62.5	12.4	2010	15	15
Åsarna	Sweden	62.7	14.4	1996	16	16
Flatruet	Sweden	62.7	12.7	2010	16	16
Ljungris	Sweden	62.9	12.7	2010	15	15
Glen	Sweden	62.9	13.6	2010	15	15
Kramfors	Sweden	63.0	18.5	1996, 2010	23	23
Vålådalen	Sweden	63.1	13.0	2010	14	14
Storulvån	Sweden	63.2	12.4	2010	19	19
Faxälven	Sweden	63.2	16.8	2000	21	20
Mårdsund	Sweden	63.2	14.0	2010	9	9
Ånn	Sweden	63.4	12.6	1996	10	10
Tångböle	Sweden	63.4	12.6	2005	9	9
Nordhallen	Sweden	63.5	12.8	2005	13	12
Nordmaling	Sweden	63.5	19.7	1996, 2010	25	25

Anjan	Sweden	63.7	12.5	2010	10	10
Yxskaftkälen	Sweden	63.8	15.2	2010	13	13
Tvärån	Sweden	63.9	19.8	2010	10	10
Hotagen	Sweden	64.0	14.8	1997	10	10
Northern migratory phenotype					192	191
Kallax	Sweden	65.5	22.1	2001	9	9
Ammarnäs	Sweden	66.0	16.1	1996	9	9
Kukkola	Sweden	66.0	24.0	1997	10	10
Gällivare	Sweden	67.2	20.8	1997	8	7
Altajärvi	Sweden	67.8	20.5	2001	11	11
Krokvik	Sweden	68.0	20.0	2001	10	10
Kaisepakte	Sweden	68.3	19.3	2001	10	10
Stordalen	Sweden	68.3	19.1	2001	20	20
Tornehamn	Sweden	68.4	18.6	2001	10	10
Kjöllefjord	Norway	70.9	27.4	2006	10	10
Tana Bru	Norway	70.2	28.2	2006	10	10
Lurio	Finland	67.2	27.6	2003	10	10
Maula	Finland	65.9	24.8	2003	10	10
Kalajoki	Finland	64.3	24.1	2003	10	10
Rutakoski	Finland	62.2	26.5	2003	15	15
Pesu	Finland	61.0	28.2	2003	10	10
Oittila	Finland	60.3	23.8	2003	10	10
Nigula	Estonia	58.1	24.9	2005	10	10
Polish-Lithuanian hybrid zone					106	106
Bialowieza	Poland	52.7	23.5	2004	18	18
Goleniow	Poland	53.6	14.7	2004	23	23
Luknajno	Poland	53.8	21.9	2004	20	20
Gdansk	Poland	54.3	18.9	2004	15	15
Voke	Lithuania	54.6	25.2	2002, 2004	30	30
Other sites					8	8
Yekaterinburg	Russia	56.9	60.7	2002	4	4
Anadyr	Russia	63.6	176.9	2005	4	4
Total					1119	1108

Table S3. Frequency of northern haplotype for each differentiated chromosome region and the mean of phenotypic measurements per sampling site. The values for each site has been calculated by only including males (N=1,029).

site	region	genos	chr1	chr3	chr5	nitrogen		size		color	
		N	N freq	N freq	N freq	N	mean	N	mean	N	mean
Stensoffa	West	9	0	0	0.06	9	7.19	9	-0.18	9	5.22
Haganäs	West	15	0	0	0.1	15	7.75	15	-0.38	0	N/A
Gräsmarö	West	13	0.04	0.04	0.15	13	7.01	13	-0.29	4	6.75
Tåkern	West	10	0	0	0.05	10	7.2	10	0.08	0	N/A
Kvismaren	West	8	0	0	0.13	8	6.34	8	-0.63	0	N/A
Smedstorp	West	10	0	0	0.15	10	7.53	10	-0.03	10	6
Orböle	West	9	0.06	0	0.28	9	7.64	9	0.09	0	N/A
Aspberget	West	7	0.14	0.07	0.21	7	8.39	7	-0.64	7	6.86
Varbergsviken	West	14	0.11	0	0.46	14	7.73	14	0.16	14	5
Bunkris	West	10	0.15	0.2	0.25	10	8.16	10	-1.62	0	N/A
Västbacka	West	14	0.14	0.04	0.25	14	8.34	14	-0.71	14	5.86
Klångstavallen	West	24	0.25	0.06	0.54	24	8.73	24	-0.11	24	5.33
Mörtsjön	West	71	0.25	0.07	0.46	71	7.92	71	0.3	71	5.06
Hudiksvall	West	9	0.22	0	0.44	9	8.52	9	0.63	0	N/A
Tanna	West	42	0.15	0.02	0.38	42	7.59	42	0.5	41	5.51
Idre	West	13	0.12	0.15	0.23	13	8.89	13	-0.26	3	4.67
Uftberget	West	19	0.21	0.18	0.24	19	8.17	19	-0.36	9	5.11
Bäcksand	West	33	0.3	0.06	0.42	33	7.9	33	0.05	33	4.18
Gnarp	West	11	0.36	0	0.64	11	8.06	11	0.24	0	N/A
Lofsdalen	West	15	0.2	0.23	0.2	15	7.12	15	0.12	15	5.87
Sörvattnet	West	10	0.2	0.3	0.25	10	6.42	10	-0.13	10	5.8
Björkön	West	22	0.45	0.11	0.52	22	8.77	22	0.03	22	5.86
Gimsjön	West	6	0.25	0.17	0.33	6	8.42	6	-0.67	6	5.67
Sundsvall	West	26	0.6	0.19	0.62	26	8.6	26	0.79	16	5.75
Alnö	West	11	0.45	0	0.55	11	8.88	11	-0.36	11	4.27
Vemdalsskälet	West	10	0.2	0.25	0.25	10	6.96	10	0.01	10	4.7
Rätan	West	13	0.27	0.15	0.42	13	9.01	13	-0.64	13	4.85
Midlanda	West	15	0.6	0.2	0.63	15	8.29	15	1.31	15	5.27
Tänndalen	West	15	0.2	0.7	0.3	15	8.89	15	-0.68	15	4.33
Åsarna	West	16	0.56	0.47	0.53	16	8.54	16	0.3	0	NA
Flatruet	West	15	0.27	0.6	0.27	15	7.58	15	-0.77	15	5.2
Ljungris	West	14	0.57	0.64	0.57	14	7.83	14	-0.48	14	5.36
Glen	West	15	0.47	0.57	0.43	15	8.01	15	-0.12	15	5.13
Kramfors	West	23	0.74	0.13	0.76	23	9.32	23	0.2	10	4.1
Vålådalen	West	14	0.46	0.32	0.39	13	7.54	14	0.06	14	5.21
Storulvån	West	17	0.41	0.79	0.35	17	9	17	-0.41	17	4.94

Faxälven	West	19	0.92	0.45	0.82	19	10	19	0.42	19	6.32
Mårdsund	West	9	0.28	0.28	0.56	9	8.4	9	-0.03	9	4.78
Ånn	West	10	0.45	0.55	0.6	10	10.16	10	0.5	0	N/A
Tångböle	West	9	0.67	0.33	0.56	9	9.35	9	0.32	9	6.44
Nordhallen	West	10	0.7	0.5	0.65	10	10.52	10	1.17	10	6.2
Nordmaling	West	25	0.94	0.38	0.82	25	10.22	25	0.15	10	4.8
Anjan	West	10	0.5	0.5	0.65	10	9.74	10	-0.58	10	4.9
Yxskaftkälen	West	13	0.81	0.5	0.73	13	10.03	13	0.02	13	5.23
Tvärån	West	6	1	0.42	0.92	6	9.3	6	0.32	6	4.83
Hotagen	West	14	0.79	0.54	0.71	14	9.35	14	0.39	4	5
Kallax	West	9	0.89	0.33	0.94	9	11.55	9	0.83	9	5.33
Ammarnäs	West	9	1	0.83	0.78	9	9.95	9	0.58	0	N/A
Kukkola	West	10	1	0.3	0.95	10	10.61	10	0.07	0	N/A
Gällivare	West	7	0.93	0.93	0.86	7	9.65	7	-0.14	0	N/A
Altajärvi	West	9	1	0.89	0.89	9	9.16	9	1.61	9	6.33
Krokvik	West	10	0.9	1	0.95	10	10.66	10	0.9	10	6.9
Kaisepakte	West	7	0.93	0.93	0.93	7	9.5	7	1.08	7	8
Stordalen	West	19	0.89	0.95	0.89	19	9.91	19	0.6	19	6.79
Tornehamn	West	10	0.9	0.8	0.85	10	9.16	10	0.22	10	6.8
Kjöllefjord	East	10	0.7	0.85	0.85	10	8.77	10	1.03	10	6.2
Tana Bru	East	10	0.95	0.85	0.95	10	9.66	10	0.41	10	5.3
Lurio	East	9	1	0.72	0.83	9	10.34	9	1.74	9	7
Maula	East	9	1	0.61	0.94	9	10.89	9	1.05	9	6.78
Kalajoki	East	9	1	0.06	1	9	10.26	9	0.54	9	5.56
Rutakoski	East	15	0.9	0.13	0.97	15	9.98	15	0.49	15	5.2
Pesu	East	9	1	0	0.89	9	10.63	9	0.34	9	5.22
Oittila	East	10	1	0.05	0.95	10	10.93	10	0	10	4.6
Nigula	East	10	0.9	0	0.9	10	9.48	10	0.68	10	5.8
Bialowieza	East	17	0.32	0	0.53	17	8.61	17	0.18	16	5.5
Goleniow	East	20	0	0.03	0.13	20	7.72	20	0.05	20	5.2
Luknajno	East	20	0.3	0.05	0.25	20	8.26	20	0.31	20	4.45
Gdansk	East	15	0.03	0	0	15	7.87	15	-0.1	15	4.87
Voke	East	30	0.78	0	0.85	30	9.62	30	0.18	30	6.07
Gadevang_Mose	East	11	0	0	0	11	6.87	11	-0.1	11	4.36
Kasted_Mose	East	12	0	0	0	12	6.95	12	0.61	12	3.83

Table S4. Highly differentiated missense mutations and inframe deletions detected in the three divergent chromosome region and all highly differentiated SNPs and indels located on scaffolds outside of the divergent regions.

Scaffold (ZF genome alignment)	Position	FST	Alleles	Gene annotation
**Div region chromosome 1**				
scaffold_23	4629356	0.88	G/T (Ser/Tyr)	missense mutation, transcription factor IIIA (GTF3A)
scaffold_23	7251678	0.88	G/A (Gly/Ser)	missense mutation, lambda-crystallin homolog (CRYL1)
scaffold_65	2669955	0.84	AGG/ - (Pro/-)	inframe deletion, retinoblastoma-associated protein (RB1)
scaffold_65	3592398-3592399	0.86	TG/CA (Gln/Trp)	missense mutation, spermatid-associated protein (SPERT)
**Div region chromosome 3**				
scaffold_6	5566344	0.79	C/T (Asp/Asn)	missense mutation, mitochondrial fission regulator 2 (MTFR2)
scaffold_6	6844616	0.77	C/T (Gly/Ser)	missense mutation, beta-taxilin (TXLNB)
**Div region chromosome 5**				
scaffold_284	61128	0.78	G/A (Ser/Asn)	missense mutation, gamma-butyrobetaine dioxygenase (BBOX1)
scaffold_284	542002	0.73	T/C (Val/Ala)	missense mutation, putative S-adenosyl-L-methionine-dependent methyltransferase METT5D1 (METTL15)
scaffold_285	329390	0.76	A/G (Ile/Thr)	missense mutation, coiled-coil domain-containing protein 73 (CCDC73)
scaffold_285	588972	1	A/C (Ile/Leu)	missense mutation, homeodomain-interacting protein kinase 3 (HIPK3)
scaffold_342	2994	0.85	T/G (Lys/Asn)	missense mutation, olfactomedin-like 1 (OLFML1)
scaffold_342	99991	0.85	G/C (Asp/Glu)	missense mutation, synaptotagmin IX (SYT9)
scaffold_342	493440	0.93	T/C (Met/Val)	missense mutation, inositol 1,4,5-trisphosphate receptor-interacting protein-like 1 (ITPRIPL1)

scaffold_342	493745	0.7	T/C (Lys/Arg)	missense mutation, inositol 1,4,5-trisphosphate receptor-interacting protein-like 1 (ITPRIPL1)
scaffold_412	22683	0.94	G/C (Glu/Asp)	missense mutation, NADH-cytochrome b5 reductase 2 (CYB5R2)
scaffold_412	293000	0.86	T/C (Asn/His)	missense mutation, uncharacterized fatty acid desaturase gene (Uniprot accession: F1NTW8)
scaffold_412	320752	0.73	G/C (Leu/Phe)	missense mutation, uncharacterized fatty acid desaturase gene (Uniprot accession: F1NBL9)
scaffold_412	353924	0.94	T/C (Phe/Ser)	missense mutation, fatty acid desaturase 2 (FADS2)
scaffold_412	415054	0.72	T/C (Thr/Ala)	missense mutation, olfactory receptor 1019 (OLFR1019)
scaffold_412	415594	0.75	G/C (Gln/Lys)	missense mutation, olfactory receptor 1019 (OLFR1019)
scaffold_412	421242	0.71	A/C (Ile/Met)	missense mutation, olfactory receptor 4Q2 (OR4Q2)
scaffold_412	425277	0.93	A/G (Leu/Pro)	missense mutation, olfactory receptor 4B1 (OR4B1)
**Rest of genome**				
scaffold_9 (chr1:89.7-97.3 Mb)	6671566	0.73	T/TTGTTTG/TTTTTTG	intron variant, limbic system-associated membrane protein (LSAMP)
scaffold_270 (chr1:99.8-100.7 Mb)	40851	0.73	C/T	176 kb from N(6)-adenine-specific DNA methyltransferase 1 (N6AMT1)
scaffold_304 (chr2:1.4-2.2 Mb)	135511	0.7	C/G	114 kb from an Augustus-predicted gene containing a coiled coin domain
scaffold_13 (chr2:8.2-19.5 Mb)	681518	0.72	A/G	intron variant, KIAA1217
scaffold_12 (chr2:49.9-60.9 Mb)	7003980	0.7	C/T	29 kb from ubiquitin-conjugating enzyme E2E 1 (UBE2E1)
scaffold_29 (chr2:117.0-124.5 Mb)	332662	0.74	A/C	intron variant, short-chain dehydrogenase/reductas

				e family 16C member 5 (SDR16C5)
scaffold_296 (chr2:150.2-151.0 Mb)	754696	0.72	A/AT	93 kb from an Augustus-predicted gene containing a coiled coin domain
scaffold_80 (chr2:151.5-154.7 Mb)	2139772	0.71	A/T	157 kb from an Augustus-predicted gene containing a coiled coin domain
scaffold_62 (chr3:78.0-81.8 Mb)	243522	0.76	A/AT	intron variant, elongation of very long chain fatty acids protein 4 (ELOVL4)
scaffold_44 (chr3:103.2-108.3 Mb)	239895	0.74	C/T	58 kb from 5'-Nucleotidase, Cytosolic IB (NT5CB1)
scaffold_150 (chr4:0.5-2.4 Mb)	639323	0.7	A/AAGAG/AAGAGA	2.7 kb from amphiregulin precursor (AREG)
scaffold_150 (chr4:0.5-2.4 Mb)	639917	0.72	C/G	2.2 kb from amphiregulin precursor (AREG)
scaffold_150 (chr4:0.5-2.4 Mb)	639919	0.71	C/T	2.2 kb from amphiregulin precursor (AREG)
scaffold_139 (chr4:10.4-12.4 Mb)	613249	0.71	C/T	72 kb from endothelin-1 receptor precursor (EDNRA)
scaffold_159 (chr4:12.4-13.7 Mb)	488222	0.73	A/T	48 kb from AF4/FMR2 family, member 1 (AFF1)
scaffold_50 (chr4:26.7-31.4 Mb)	4719902	0.71	A/T/AT	174 kb from membrane-associated ring finger (C3HC4) 1 (MARCH1)
scaffold_426 (chr4A:19.9-20.3 Mb)	182071	0.75	C/T	intron variant, mediator complex subunit 12 (MED12)
scaffold_395 (chr5:6.9-7.4 Mb)	75287	0.71	A/T	2.5 kb from BTB/POZ domain-containing protein 18 (BTBD18)
scaffold_113 (chr5:8.2-10.5 Mb)	866053	0.84	A/G	intron variant, signal peptide, CUB domain, EGF-like 2 (SCUBE2)
scaffold_10 (chr7:7.2-18.3 Mb)	10490122	0.71	A/C	300 kb from Contactin-associated protein-like 5 (CNTNAP5)
scaffold_235 (chr11:10.4-11.2 Mb)	544362	0.7	C/T	intron variant, junctophilin 3 (JPH3)
scaffold_190 (chr12:20.1-21.6 Mb)	286527	0.8	A/G	33 kb from ER degradation-enhancing alpha-mannosidase-like 1 (EDEM1)
scaffold_51 (chr13:1.8-6.6 Mb)	2270593	0.86	A/C	intron variant, transcription factor COE1 (EBF1)

scaffold_76 (chr14:2.6-5.3 Mb)	615752	0.75	C/T	intron variant, GSG1-like (GSG1L)
scaffold_46 (chr14:10.5-15.4 Mb)	2868420	0.72	A/G	58 kb from UNC homeobox (UNCX)
scaffold_127 (chrZ:64.3-65.9 Mb)	1810236	0.72	A/G	intron variant, COBW domain-containing protein 1 (CBWD1)
scaffold_97 (no mapping)	1716335	0.72	A/G	25 kb from methyltransferase like 11B (METTL11B)
scaffold_1563 (no mapping)	3173	0.76	G/T	no annotation on scaffold
scaffold_1909 (no mapping)	9682	0.76	G/A	no annotation on scaffold
scaffold_1909 (no mapping)	9801	0.74	C/T	no annotation on scaffold
scaffold_2121 (no mapping)	1566	0.72	A/C	no annotation on scaffold
scaffold_3905 (no mapping)	1172	0.78	C/T	no annotation on scaffold
scaffold_3905 (no mapping)	1320	0.75	A/G	no annotation on scaffold

Table S5. Results of correlation analyses between mean phenotypic trait estimates and differentiated chromosome region northern haplotype frequency per sampling site. Reported are correlation coefficient, 95 % confidence interval of correlation coefficient, p value and R^2.

trait	chrom region	region	corr coeff	95CI low	95CI high	p value	R^2
mean nitrogen	chr1	all sites	0.844	0.760	0.900	<2E-16	0.712
mean nitrogen	chr1	West Baltic	0.791	0.665	0.873	6.63E-13	0.626
mean nitrogen	chr1	East Baltic	0.951	0.862	0.983	1.51E-08	0.905
mean nitrogen	chr3	all sites	0.439	0.229	0.610	1.30E-04	0.193
mean nitrogen	chr3	West Baltic	0.545	0.326	0.708	1.72E-05	0.297
mean nitrogen	chr3	East Baltic	0.315	-0.214	0.701	2.35E-01	0.099
mean nitrogen	chr5	all sites	0.847	0.764	0.902	<2E-16	0.717
mean nitrogen	chr5	West Baltic	0.814	0.701	0.888	3.93E-14	0.663
mean nitrogen	chr5	East Baltic	0.915	0.767	0.970	6.95E-07	0.837
mean size	chr1	all sites	0.562	0.378	0.703	0.000	0.316
mean size	chr1	West Baltic	0.563	0.351	0.721	0.000	0.317
mean size	chr1	East Baltic	0.495	-0.001	0.795	0.051	0.245
mean size	chr3	all sites	0.318	0.092	0.514	0.007	0.101
mean size	chr3	West Baltic	0.310	0.049	0.532	0.021	0.096
mean size	chr3	East Baltic	0.688	0.293	0.883	0.003	0.474
mean size	chr5	all sites	0.557	0.372	0.700	0.000	0.311

mean size	chr5	West Baltic	0.597	0.394	0.744	0.000	0.356
mean size	chr5	East Baltic	0.446	-0.064	0.771	0.083	0.199
mean color	chr1	all sites	0.354	0.108	0.560	5.89E-03	0.126
mean color	chr1	West Baltic	0.267	-0.036	0.525	8.32E-02	0.071
mean color	chr1	East Baltic	0.606	0.158	0.847	1.28E-02	0.368
mean color	chr3	all sites	0.446	0.214	0.630	4.01E-04	0.199
mean color	chr3	West Baltic	0.380	0.089	0.610	1.21E-02	0.144
mean color	chr3	East Baltic	0.597	0.144	0.843	1.46E-02	0.356
mean color	chr5	all sites	0.347	0.100	0.554	7.10E-03	0.120
mean color	chr5	West Baltic	0.226	-0.079	0.493	1.44E-01	0.051
mean color	chr5	East Baltic	0.627	0.190	0.856	9.35E-03	0.393

Table S6. Estimated parameters for geographical clines of northern haplotypes in each of the three differentiated chromosome regions. Measurements are in km, ML refers to maximum likelihood estimates and LL refers to log likelihood.

**locus**	**Best fitting cline model**	**center (ML)**	**lower 2LL**	**upper 2LL**	**width (ML)**	**lower 2LL**	**upper 2LL**
Sweden							
Chrom 1	no introgression tails	756	749	764	360	326	387
Chrom 3	introgression tail on right side	812	789	840	271	219	354
Chrom 5	no introgression tails	741	700	774	688	524	924
Poland							
Chrom 1	no introgression tails	590	520	624	373	257	515
Chrom 5	no introgression tails	567	509	604	249	128	366

Table S7. Statistical significance from testing differences in the mean 10 kb window estimates of genetic variation (nucleotide diversity, Tajima's D, d_{XY} and d_A) among each differentiated chromosome region and the rest of the genome. Pairwise significance between regions are derived from a Tukey's HSD test. P values are reported below the diagonal and the difference in mean estimates above the diagonal.

**Nucleotide diversity (ANOVA: df=7,183660, F=200.9, p<2e-16)**	**chrom 1 south**	**chrom 1 north**	**chrom 3 south**	**chrom 3 north**	**chrom 5 south**	**chrom 5 north**	**non-divergent genome south**	**Non-divergent genome north**
chrom 1 south		0.0016	0.0005	0.0001	0.0001	0.0011	0.0002	0.0002
chrom 1 north	<1e-7		0.0021	0.0016	0.0018	0.0006	0.0018	0.0014
chrom 3 south	0.0002	<1e-7		0.0006	0.0003	0.0016	0.0003	0.0007
chrom 3 north	1	<1e-7	0.000003		0.0002	0.001	0.0003	0.0001
chrom 5 south	0.99	<1e-7	0.34	0.88		0.0012	0.00004	0.0003
chrom 5 north	<1e-7	0.01	<1e-7	<1e-7	<1e-7		0.0013	0.0009
non-divergent genome south	0.31	<1e-7	0.002	0.02	1	<1e-7		0.0004
non-divergent genome north	0.22	<1e-7	<1e-7	0.74	0.24	<1e-7	<1e-7	

**Tajima's D (ANOVA: df=7,180976, F=830.4, p<2e-16)**	**chrom 1 south**	**chrom 1 north**	**chrom 3 south**	**chrom 3 north**	**chrom 5 south**	**chrom 5 north**	**non-divergent genome south**	**Non-divergent genome north**
chrom 1 south		0.53	0.19	0.45	0.14	0.56	0.64	0.63
chrom 1 north	<1e-7		0.34	0.08	0.66	0.03	0.12	0.11
chrom 3 south	<1e-7	<1e-7		0.26	0.32	0.37	0.45	0.45
chrom 3 north	<1e-7	0.0002	<1e-7		0.59	0.11	0.19	0.18
chrom 5 south	9E-07	<1e-7	<1e-7	<1e-7		0.69	0.78	0.77
chrom 5 north	<1e-7	0.93	<1e-7	0.0006	<1e-7		0.09	0.08
non-divergent genome south	<1e-7	<1e-7	<1e-7	<1e-7	<1e-7	0.003		0.008

non-divergent genome north	<1e-7	<1e-7	<1e-7	<1e-7	<1e-7	0.01	9e -4

**d_{XY} (ANOVA: df=3,92630, F=456.5, p<2e-16)**	**chrom 1**	**chrom 3**	**chrom 5**	**non-divergent genome**
chrom 1		0.0009	0.0005	0.0023
chrom 3	<1e-7		0.0004	0.0015
chrom 5	0.02	0.06		0.0019
non-divergent genome	<1e-7	<1e-7	<1e-7	

**d_A (ANOVA: df=3,92630, F=39623, p<2e-16)**	**chrom 1**	**chrom 3**	**chrom 5**	**non-divergent genome**
chrom 1		0.0003	0.0003	0.0008
chrom 3	<1e-7		0.0006	0.0012
chrom 5	<1e-7	<1e-7		0.0006
non-divergent genome	<1e-7	<1e-7	<1e-7	

Table S8. Annotation of the 10 most differentiated filtered 10kb windows and array SNPs in each of the divergent chromosome regions. Up- and downstream refer to the orientation relative to the transcription of the gene.

**Scaffold**	**Position**	**FST**	**Annotation**
**10 kb windows**			

Chromsome 1

scaffold_65	30001-40000	0.80	31 kb upstream of FRAS1 related extracellular matrix protein 2 (FREM2)
scaffold_65	10001-20000	0.80	51 kb upstream of FRAS1 related extracellular matrix protein 2 (FREM2)
scaffold_65	1-10000	0.79	61 kb upstream of FRAS1 related extracellular matrix protein 2 (FREM2)
scaffold_23	1-10000	0.78	36 kb downstream of Ubiquitin-fold modifier 1 (UFM1)
scaffold_65	40001-50000	0.73	21 kb upstream of FRAS1 related extracellular matrix protein 2 (FREM2)
scaffold_23	1940001-1950000	0.71	124 kb upstream of neurobeachin (NBEA)
scaffold_65	110001-120000	0.71	intron of FRAS1 related extracellular matrix protein 2 (FREM2)
scaffold_23	6420001-6430000	0.71	251.2 kb downstream of glia-activating factor (FGF9)
scaffold_23	1830001-1840000	0.69	14 kb upstream of neurobeachin (NBEA)
scaffold_23	1820001-1830000	0.67	4 kb upstream of neurobeachin (NBEA)

Chromosome 3

scaffold_6	11940001-11950000	0.62	intron of estrogen receptor (ESR1)
scaffold_6	1360001-1370000	0.62	introns and exons of ryanodine receptor 2 (cardiac) (RYR2)
scaffold_6	1160001-1170000	0.62	intron of ryanodine receptor 2 (cardiac) (RYR2)
scaffold_6	11090001-11100000	0.61	162 kb upstream of opioid receptor, mu 1 (OPRM1)
scaffold_6	7240001-7250000	0.59	343 kb upstream of cbp/p300-interacting transactivator 2 (CITED2)
scaffold_6	11100001-11110000	0.58	169 kb upstream of regulator of G-protein signaling 17 (RGS17)
scaffold_6	11980001-11990000	0.58	15 kb upstream of estrogen receptor (ESR1)
scaffold_6	7340001-7350000	0.58	443 kb upstream of cbp/p300-interacting transactivator 2 (CITED2)
scaffold_6	11330001-11340000	0.58	intron of regulator of G-protein signaling 17 (RGS17)
scaffold_6	5390001-5400000	0.58	intron of phosphodiesterase 7B (PDE7B)

Chromosome 5

scaffold_342	30001-40000	0.85	22.7 kb upstream of olfactomedin-like 1 (OLFML1)
scaffold_342	1-10000	0.84	entire olfactomedin-like 1 (OLFML1)
scaffold_342	260001-27000	0.83	intron of spondin-1 precursor (SPON1)
scaffold_342	270001-28000	0.81	intron of spondin-1 precursor (SPON1)
scaffold_342	20001-30000	0.80	12.7 kb upstream of olfactomedin-like 1 (OLFML1)
scaffold_412	340001-35000	0.79	first exons and upstream regions of Fatty acid desaturase 1 (FADS1) and Fatty acid desaturase 2 (FADS2)
scaffold_342	40001-50000	0.79	20.5 kb downstream of synaptotagmin IX (SYT9)
scaffold_412	250001-260000	0.76	exon and upstream region of ankyrin repeat and BTB (POZ) domain containing 2 (ABTB2)
scaffold_342	10001-20000	0.76	2.7 kb upstream of olfactomedin-like 1 (OLFML1)
scaffold_412	190001-200000	0.74	intron of ankyrin repeat and BTB (POZ) domain containing 2 (ABTB2)

SNP array

chromosome 1

scaffold_65	842155	0.80	32 kb downstream of Forkhead box O1 (FOXO1)
scaffold_23	5096563	0.63	missense mutation, Ring finger protein (C3H2C3 type) 6 (RNF6)

scaffold_23	2238755	0.62	38 kb upstream of StAR-related lipid transfer (START) domain containing 13 (STARD13)
scaffold_23	875447	0.61	62 kb downstream of Cyclin A1 (CCNA1)
scaffold_23	875505	0.60	62 kb downstream of Cyclin A1 (CCNA1)
scaffold_23	1312098	0.57	7 kb downstream of Serine/threonine-protein kinase DCLK1 (DCLK1)
scaffold_65	3301727	0.55	25 kb upstream of KIAA0226-like (KIAA0226L)
scaffold_65	1249264	0.54	5 kb upstream of Dehydrogenase/reductase (SDR family) member 12 (DHRS12)
scaffold_23	7824385	0.53	12 kb upstream of Rho GTPase activating protein 20 (ARHGAP20)
scaffold_65	2662724	0.52	4.8 kb downstream of regulator of chromosome condensation (RCC1) and BTB (POZ) domain containing protein 2 (RCBTB2); 1.2 kb downstream of retinoblastoma-associated protein (RB1)
chromosome 3			
scaffold_6	2468519	0.41	intron variant, Regulator of G-protein signaling 7 (RGS7)
scaffold_6	2662788	0.40	intron variant, Regulator of G-protein signaling 7 (RGS7)
scaffold_6	11468398	0.38	23 kb downstream of VIP peptides Intestinal peptide PHI-27-like Vasoactive intestinal peptide (VIP)
scaffold_6	220657	0.36	67 kb downstream of Translocase of outer mitochondrial membrane 20 homolog (yeast) (TOMM20)
scaffold_6	4705740	0.36	93 kb downstream of Epilepsy, progressive myoclonus type 2A, Lafora disease (laforin) (EPM2A)
scaffold_6	10990347	0.34	62 kb upstream of Opioid receptor, mu 1 (OPRM1)
scaffold_6	1596492	0.34	195 kb downstream of Ryanodine receptor 2 (cardiac) (RYR2)
scaffold_6	111725	0.32	4.8 kb upstream of interferon regulatory factor 2 binding protein 2 (IRF2BP2)
scaffold_6	2489451	0.31	intron variant, Regulator of G-protein signaling 7 (RGS7)
scaffold_6	1147878	0.31	intron variant, Ryanodine receptor 2 (cardiac) (RYR2)
chromosome 5			
scaffold_342	29329	0.67	22 kb upstream of Olfactomedin-like 1 (OLFML1)
scaffold_412	421242	0.62	missense mutation, Olfactory receptor 4Q2 (olfactory receptor OR14-21) (OR4Q2); 4.3 kb upstream of Proto-oncogene Mas (MAS1); 3.6 kb downstream of Olfactory receptor 4B1 (OST208) (OR4B1)
scaffold_412	421690	0.59	missense mutation, Olfactory receptor 4Q2 (olfactory receptor OR14-21) (OR4Q2); 3.8 kb upstream of Proto-oncogene Mas (MAS1); 3.2 kb downstream of Olfactory receptor 4B1 (OST208) (OR4B1)
scaffold_412	24858	0.57	synonymous variant, PTPRF interacting protein, binding protein 2 (liprin beta 2) (PPFIBP2); intronic variant, CYB5R2,NADH-cytochrome b5 reductase 2 (CYB5R2)
scaffold_412	421575	0.56	synonymous variant, Olfactory receptor 4Q2 (olfactory receptor OR14-21) (OR4Q2); 3.9 kb upstream of Proto-oncogene Mas (MAS1); 3.3 kb downstream of Olfactory receptor 4B1 (OST208) (OR4B1)
scaffold_412	376267	0.56	intron variant, Guanine nucleotide exchange factor for Rab-3A (RAB3IL1)
scaffold_342	198636	0.52	28 kb downstream of Spondin-1 precursor (SPON1)
scaffold_412	361868	0.50	intron variant, Fatty acid desaturase 2 (FADS2)
scaffold_412	142401	0.50	intron variant, Ankyrin repeat and BTB (POZ) domain containing 2 (ABTB2)
scaffold_285	772504	0.50	3' UTR variant, KIAA1549-like (KIAA1549L)

107

Table S9. Primers used for long-range PCR across the ends of the differentiated regions on chromosome 1 and 3 and their adjacent scaffolds determined from whole-genome alignments to other bird genomes (Fig S1). The gap between the end of the differentiated region of chromosome 1 and the closest upstream scaffold (scaffold 98) was determined to be too large for successful amplification and primers were therefore not designed for this genomic interval. The only pairs that resulted in PCR products of estimated size were 65_f_2 + 30_r_2 for chromosome 1 and 69_f_2 + 6_r for chromosome 3.

Name	Sequence 5’-3’	Region	Genomic position
scaffold_30_r	CAGCTGAAGAAATTGGATGCCTG	chr1: adjacent scaffold upstream	Scaffold 30:2385-2407
scaffold_30_r_2	GTGGCTGAATAACATGCACTGAC	chr1: adjacent scaffold upstream	Scaffold 30:584-606
scaffold_65_f	CACGACTAAAGCTCCCAACTTCA	chr1: differentiated region start	Scaffold 65:3671426–3671448
scaffold_65_f_2	TCTGTTCCACCTCCCTTTGCA	chr1: differentiated region start	Scaffold 65:3671185–3671205
scaffold_69_f	TGGAGAATTGCTAAGCAGGAGC	chr3: adjacent scaffold upstream	Scaffold 69:3492643–3492664
scaffold_69_f_2	TCTCCATATCAGCCTGCACAG	chr3: adjacent scaffold upstream	Scaffold 69:3493032–3493052
scaffold_6_r	TCTCGAGACCAGAGCATGTGA	chr3: differentiated region start	Scaffold 6:1552-1572
scaffold_6_r_2	ACACAGTTTGGGAGCAATGTGAAT	chr3: differentiated region start	Scaffold 6:1900-1923
scaffold_6_f	TGAGCATGTAGAAAGTATTGCTGC	chr3: differentiated region end	Scaffold 6:13126335–13126358
scaffold_6_f_2	GCCAGGGACATGTAGAGATAAG	chr3: differentiated region end	Scaffold 6:13126456–13126477
scaffold_186_r	GAGAAACACACCACCATTCAAGG	chr3: adjacent scaffold downstream	Scaffold 186:1458093-1458115
scaffold186_r_2	AGACAGCATTAAGCCTGCAGC	chr3: adjacent scaffold downstream	Scaffold 186:1457440–1457460

Supplementary methods

Automated probe design (454 transcriptome data)

The vast majority of probes (N=5,839) was automatically designed from 454 willow warbler transcriptome reads mapped to the zebra finch genome (Lundberg et al. 2013) The zebra finch genome assembly (version 3.2.4), was downloaded from the genome bioinformatics website of University of California at Santa Cruz (UCSC) (www.genome.ucsc.edu). The genome assembly consists of 35 chromosomes or linkage groups, the mitochondrial genome, 33 random chromosomes consisting of concatenated sequences that have been associated with a particular chromosome, but not successfully ordered within it, and finally a chromosome (ChrUn) containing sequences that have not been linked to any particular chromosome or linkage group.

The willow warbler transcriptome sequence data consists of 1.8 million sequence reads originating from two libraries of pooled cDNA from eight brain tissue samples of each migratory phenotype, respectively. For further details on samples, cDNA library preparation and sequencing, see Lundberg *et al.* (2013). In order to potentially improve the mapping of reads to the zebra finch genome, we used a newer version (2013-09-19) of GMAP(Wu & Watanabe 2005) compared to the earlier study. We used default settings with the exception of cross-species mapping (--cross-species flag) and to output alignments into a Sequence Alignment/Mapping (SAM) format (Li et al. 2009), and mapped reads from each migratory phenotype separately.

The SAM files were subsequently processed with samtools version 0.1.19 (Li et al. 2009). They were first converted into Binary Alignment/Mapping (BAM) files using the view command, then sorted and indexed based on the zebra finch genome, using the sort and index commands, respectively. The mpileup command was used to generate a pileup file containing

nucleotide data for each aligned position in the two migratory phenotype bam files. For the command we excluded reads with a mapping quality score below 40.

A customized Perl script was used to retrieve only the variable positions within the willow warbler sequence pools (i.e. excluding fixed differences between the zebra finch and the willow warbler) and to convert the nucleotide and indel information to a format more suitable for downstream analyses. Another customized Perl script was used to call a consensus genotype at every variable position and length polymorphisms in the data set based on the minor allele count (MAC) and the minor allele frequency (MAF). For both SNPs and length polymorphisms we set MAC≥3 and MAF≥0.05. Similarly, insertions or deletions between the willow warbler and the zebra finch genome were considered fixed if the minor length polymorphism had a frequency or count below the two thresholds.

From the output we extracted positions containing bi-allelic SNPs with no significant length polymorphism detected at the same position. Another customized Perl script was used to extract the consensus sequence of a maximum of 60 bp on each side of the SNP in the modified pileup file. This preliminary set of probes was mapped back to the genome using the GMAP software with the same parameters as before. This raw set was filtered for probes that had at least 50 bp of contiguous sequence on either side of the SNP, an A or T content of maximum 50 %, a minimum sequencing depth of at least two reads at any position in the sequence, no ambiguous bases (Ns) or significant indels, a maximum of three heterozygous positions over the entire sequence, no heterozygous positions within the five nucleotides immediately surrounding the target SNP and the best mapping located at the expected position in the zebra finch genome. 8,376 probes were obtained after filtering and submitted to Illumina’s online Assay Design Tool

(ADT). Of 7,772 probes that did not receive any error code in the ADT tool, we selected the 5,850 with the highest design scores (mean=0.98, sd=0.02).

An additional four automatically generated probes were included into the final array design, despite not passing all of the filtering requirements. These probes were located close to the genes cryptochrome 4 (CRY4), period homolog 2 (PER2), Aryl hydrocarbon receptor nuclear translocator-like protein 1 (ARNTL) and Adenylate cyclase activating polypeptide 1 (ADCYAP1), which are involved in circadian timekeeping. These genes could potentially be related to seasonal timing and hence various timing aspects of migratory behavior.

RAD data for array design

Ninety-one probes were manually designed from willow warbler Restriction-associated DNA (RAD) sequence data mapped to the zebra finch genome. This data set originated from 10 samples from each subspecies (Table S1). RAD libraries were produced according to an optimized protocol based on Amores *et al.* (2011) and Etter *et al.* (2011). One µg of high molecular weight, RNA free genomic DNA was digested with 20 U/µl restriction enzyme Sbfi-HF (New England Biolabs: R3642). Adapter P1, which contains forward amplification and Illumina sequencing primer sites, was ligated to the complementary ends of the fragments. This adapter also contains a seven basepair sample-specific bar code that differed at least three positions between samples to avoid false assignment due to sequencing errors. The individually barcoded samples were multiplexed in order to minimize costs and differences in amplification efficiency that may arise between different library preparations. Multiplexed samples were randomly sheared by sonication on a Bioruptor Standard UCD-200 (Diagenode, Belgium) to an average size of 300-600 bp, creating a library of P1 restriction site-ligated molecules (removing free unligated or concatemerised P1

adapters) with random variable ends for amplification. Following size selection by electrophoresis, purified DNA was ligated to a second “Y” P2 adapter with divergent ends containing a 3’ dT overhang onto the ends of blunt DNA fragments with 3’ dA overhangs. This adapter design ensures that only P1 adapter-ligated RADtags are amplified during the final PCR, as the reverse amplification primer can only bind to P2 if the complementary sequence is filled in during the first elongation step that originates from P1 amplification primer. To enrich P1 and P2 adapter-ligated DNA fragments we used high fidelity PCR amplification. DNA quantification was carried out using Qubit® 2.0 Fluorometer (Invitrogen, Carlsbad, CA, USA) with final concentrations between 7.76 and 19.8 ng/µl. The libraries were sequenced on a Highseq2000 (Illumina,, CA, USA).

The resulting sequence reads were first inspected in FastQC v. 0.10.1 to determine the base quality distribution of reads. Stacks version 1.08 (Catchen et al. 2013) was then used to filter away low quality reads, de-multiplex reads of different individuals in the same library and to remove potential PCR clones. The trimmed reads were then mapped to the genome of the zebra finch using Bowtie2 (Langmead & Salzberg 2012). We used default settings of the software with the exception of allowing a maximum of one mismatch in a seed alignment (-N 1), a minimum and maximum mismatch penalty of 1 and 2, respectively (-mp 2,1), a minimum fragment length for paired-end alignments of 150 bp (-I 150) and a maximum fragment length for paired-end alignments of 1000 bp (-X 1000). Only concordant alignments with predicted read orientation and where both of the reads mapped to unique locations were included (-no-mix,-no-discordant).

We used Stacks version 1.08 (Catchen et al. 2013) to call genotypes from the mapped sequence data. For this purpose we only used the first read in each pair, which contained the restriction site. In the software we used the ref_map.pl script to perform genotype calling and

subsequently calculate population genetics statistics. For the script we used default settings with the exception of requiring a minimum depth of four reads to report a stack and a minimum of 50 % of genotyped samples in the population to process a locus. Genetic differentiation was estimated using F_{ST} and Fisher's exact test for significance.

As a complement to Stacks, SNPs were also called using Freebayes version 9.9.2 (Garrison & Marth 2012). The software was used with default settings with the exception of requiring a minimum 50 reads for each site, using standard filters and reporting no complex alleles. The output was filtered using vcftools version 0.1.11 (Danecek et al. 2011) using a minimum of five reads for an individual genotype to be called. The same software was used to calculate genetic differentiation. A customized Perl script was used to further filter the SNP genotypes bases on a minimum of five individuals for each genotype and a MAF≥0.05. Finally, we used Fisher's exact test in R to obtain locus-specific p values for allele-frequency differences between each migratory phenotype group. Given a limited number of highly differentiated SNPs in each of the data sets, we also included SNPs with comparatively low sequencing depth or that were only genotyped in a fraction of the samples.

Manually designed probes

The array further covered 91 manually designed probes originating from the RAD sequence data and 34 probes derived from Sanger sequence analyses in three earlier detected divergent chromosome regions between the two subspecies of the willow warbler (Lundberg et al. 2013). In all cases probe design was performed in Geneious Pro version 5.6.6 (Drummond et al. 2012) using alignments from previously generated Sanger sequence data or from local extracted regions from BAM files. In some cases the alignment surrounding the targeted SNP contained

SNPs with a low minor allele frequency. In order for the probe sequence to be successfully submitted to the ADT, these variable positions were masked with the most common allele. This would have the consequence of a probe sequence that could be successfully hybridized to most samples, at the cost of potentially losing genotypes of samples containing the rarer and mismatching allele in the probe sequence. When a final set of automatically and manually designed probes had been generated, 25 probes were chosen at random in order to serve as replicates. In addition, a manually designed probe sequence had been unintentionally replicated as it was already included in the automatically generated data set.

SNP array samples and hybridization

Samples were primarily collected from two hybrid zones between the migratory phenotypes, one located in central Sweden (N=702) and the second in Poland/Lithuania (N=106). We also included reference samples for the southern migratory phenotype from southern Scandinavia and Scotland (N=111) and for the northern migratory phenotype from Northern Scandinavia, Finland and Estonia (N=192) (Table S2). Four samples each were included from Yekaterinburg and Anadyr in Russia. The samples from Anadyr were collected within the breeding distribution of the subspecies, *P.t.yakutensis* (Cramp 1992).

Birds in their second calendar year or older had been collected between 1996 and 2010 by playback-assisted catching with mist nets. Detailed description on handling of birds and extraction of DNA are given in Bensch *et al.* (1999). Samples from Anadyr originate from muscle tissue, DNA was extracted using a lysis buffer protocol (Laird et al. 1991). All samples were diluted to a concentration of 50-90 ng/µl, following a quantification of DNA content and purity in a Nanodrop 8000 (Thermo Scientific, Wilmington, Delaware, USA).

Hybridization of samples to arrays was performed at SCIBLU, Lund, Sweden following the Infinium® HD Assay Ultra Protocol guide available at http://www.support.illumina.com/documentation.html. In order to avoid spurious population structure originating from batch effects, samples from different sites were randomized over a total of twelve 96-plates. Normalization of hybridization intensities across plates was aided by adding three samples that worked well on the first plate onto each of the following eleven plates.

SNP array data processing

The raw array data was converted into genotype data using Genome studio version 2011.1 (Illumina, CA, USA). The Genome studio data was converted into a ped and map format and imported into PLINK version 1.07 (Purcell et al. 2007) for quality trimming and conversion into formats suitable for downstream analyses. PLINK requires that sex and non-autosomal chromosomes are encoded with specific integers, which is dependent on the number of chromosomes of the species. The used version was not built to handle data from avian genomes, but we overcame this problem by using settings for the dog genome, which has a similar number of chromosomes to the zebra finch genome, 2n=78 and 2n=80, respectively (Lindblad-Toh et al. 2005, Pigozzi & Solari 1998) . The Z chromosome was designated the same number (37) as the X chromosome in the dog. In the software we removed samples with a genotype call rate of less than 95%, SNPs with a minor allele frequency of less than 0.01 and SNPs with a maximum of 5% missing data. Most probes were excluded due to a low minor allele frequency. Once the willow warbler genome assembly had been completed, we mapped the probe sequences to the willow warbler genome using GMAP (Wu & Watanabe 2005), and located the position of the SNPs in the alignments. Only filtered SNPs with their probe sequence successfully mapped to the genome were

included in downstream analyses. One highly differentiated SNP that was designed from alignments on chromosome 3 in the zebra finch was found to have its genotypes more correlated with those of SNPs on chromosome 5. In the willow warbler assembly the probe sequence of this SNP mapped to a scaffold located on chromosome 5, which contained other highly differentiated SNPs. Due to the discrepancy in location between the species, this SNP was removed from downstream analyses. The final filtered data set was comprised of 4,063 SNPs that were enriched for a high minor allele frequency (mean=0.19).

SNP array population genetics

We used hierfstat (Goudet 2005) to calculate genetic differentiation between 111 southern and 191 northern migratory phenotypes and investigated population structure across all sampling sites using a principal component analysis (PCA) implemented in *smartpca* function in the Eigensoft package version 5.0.1 (Patterson et al. 2006). We ran the PCA using both all SNPs and only those outside of the divergent chromosome region. In order to run the software we needed SNP locations on chromosomes and genetic distances estimated between them. For this purpose we used the positions of the SNPs on chromosomes of the zebra finch, which had been inferred during the array design, when transcriptome and RAD data had been mapped to the zebra finch genome. As genetic distance, we used a constant rate of 3.0 cM/Mb, which had been estimated as a genome-wide average in the collared flycatcher using a high-density linkage map (Kawakami et al. 2014). We also estimated isolation by distance (IBD) between sample sites in Genepop version 4.4.2 (Rousset 2008) by fitting a regression line between standardized differentiation (F_{ST}/(1-F_{ST}) across log(distance). The strength of IBD, as quantified by the slope, was provided a confidence interval using bootstrapping. When including all SNP genotypes, the slope was

0.0054/log(km) (95CI:0.0045-0.0065) and when excluding SNPs within the differentiated chromosome regions, isolation by distance decreased by an order of magnitude to 0.00071/log(km) (95CI:0.00038-0.0011).

***de novo* genome sequencing**

DNA from a single male caught in Stordalen, Northern Sweden (68.3°N, 19.1°E) in 2001 was used for *de novo* genome sequencing. This sample was chosen because it was homozygous (northern) for three previously identified divergent chromosome regions (Lundberg et al. 2011, Lundberg et al. 2013) and had worked well in a previous preparation of a restriction-associated DNA (RAD) library (this paper). We extracted DNA from blood using a phenol-chloroform protocol(Sambrook et al. 1989). Prior to the construction of sequence libraries we quantified the concentration of sample with a Qubit 2.0 (Invitrogen, Carlsbad, CA, USA) and further checked the quality on a 1% agarose gel, on which it showed little fragmentation and a clear band > 10kb. Preparation of four sequence libraries was performed at ScilifeLab, Stockholm, Sweden, with targeted insert sizes of 180 bp, 650 bp, 3 kb and 7 kb, respectively. The preparation of the short insert libraries (180 and 650 bp) was done following the TruSeq DNA PCR free protocol (Illumina, CA, USA) according to the manufacturer's instruction, with the exception of shearing 1.1 g of gDNA to approximately 180 using a Covaris S2 instrument (Covaris, Inc, MA, USA), using an Agilent NGS workstation (Agilent, CA, USA) for automation, removing the fragmentation clean up and replacing the size selection step with customized magnetic bead clean-up methods (Borgstrom et al. 2011). The preparation of mate pairs (3kb and 6kb) followed the Nextera Mate Pair Sample Preparation (Illumina, CA, USA) gel-free according to the manufacturer's instructions but with library clean up automated and performed on a MBS 1200

pipetting station (Nordiag AB, Sweden) using magnetic bead clean-up methods (Lundin et al. 2010). All of the libraries were sequenced on a Hiseq 2500 (Illumina, CA, USA) at ScilifeLab, Stockholm, Sweden. For the 650 bp library we used v4 chemistry, which resulted in a larger number of sequences per lane and longer reads. The raw sequence data was processed with trimmomatic-0.30 (Bolger et al. 2014) in order to remove adapter and linker sequences and to trim low quality bases. The quality of the processed reads was verified by using FastQC version 0.10.01 (Andrews 2010). After filtering we obtained 147 Gb of sequence, which assuming a genome size of 1.2 Gb as of the zebra finch (Warren et al. 2010), would correspond to an expected coverage of 123x.

***de novo* genome assembly**

The trimmed sequence data was assembled using ALLPATHS-LG release 49618 (Gnerre et al. 2011). The assembly was more fragmented than expected from a bird genome with the available amount of sequence data (25,655 scaffolds, N50=190kb), which is likely explained by the high level of sequence diversity in willow warbler (Bensch et al. 2006b). To account for this problem, we performed another assembly with ALLPATHS-LG using the HAPLOIDIFY=true setting. This setting increased scaffold size by an order of magnitude (N50=3.5 Mb) and reduced the number of scaffolds to 6,000. The number of contigs for this assembly was 38,092 and the contig N50 length 114 kb. To further improve the continuity of the assembly, we used Gapcloser version 1.12 from the SOAPdenovo package (Li et al. 2010). We used the two paired-end libraries (180 and 650 bp) and the genome assembly as input and ran the software with default settings, except for increasing the maximum allowed read length to 125 bp. The software was able to reduce the number of gaps from 32,102 to 16,241 and the number of nucleotides in gaps

from 38.5 million to 17.7 million. To independently validate if the gap closing had improved the assembly, we used CEGMA version 2.5 (Parra et al. 2009) to map 258 ultraconservative genes among eukaryotes onto the raw and gap closed assembly. The gap closed version showed a higher number of both complete (148), i.e. alignments covering at least 70 % of the gene, and partial genes (215), i.e. genes with alignments above a score specific to each gene, than the original assembly (143 complete, 205 partial), and was chosen for downstream analyses. As expected, the number of complete genes is considerably lower than what was found in the more well-assembled chicken genome (version 2.1, N=208), but the total number of genes in chicken (N=212) is almost identical to what we found in the willow warbler genome (Parra et al. 2009)

In order to remove potential contaminant sequence data from the assembly we divided scaffolds into sequence chunks of maximum 10kb and used blastn to search them against the zebra finch genome, the chicken genome and to the NCBI nt database (downloaded October 2014). Sequence data from both bird species was downloaded from ENSEMBL (www.ensembl.org) and did not include random chromosomes (scaffolds assigned to particular chromosomes or linkage groups, but not successfully ordered within them) or scaffolds that have not been assigned to any chromosome or linkage group (ChrUn). Of the total 110,658 chunks, 107,939 had a hit in at least one of the three data bases. The vast majority of chunks had the highest alignment score in the zebra finch or chicken genome and only 5,232 chunks had a higher score in the nt database, or alignments absent in the other data bases. Of the chunks scoring highest in the nt database, the majority had a best hit to a sequence from a bird. Hits to other vertebrates were almost exclusively restricted to sequences with shorter alignments (<1000 bp) and were not considered sufficient evidence of contamination. However, two scaffolds

showed long alignments and high identity to sequences from *E. coli* and a cloning vector, respectively. These scaffolds were removed from further analyses.

To arrange the scaffolds in their putative order along chromosomes in the willow warbler genome, we used SatsumaSynteny version 2.0 2015-05-24 (Grabherr et al. 2010) with default settings to align scaffolds to the genome of the zebra finch (version 3.2.24), collared flycatcher (version 1.5) and chicken (version 4.0). As with the contamination check, we excluded chromosomes comprised of randomly arranged or unplaced scaffolds. Three scaffolds mapping to the mitochondrion were further assembled by aligning them to the full mitochondrial genome sequence of the yellow-browed warbler *Phylloscopus inornatus* (Qing et al. 2015). The scaffolds partly overlapped each other, but between two of them, the overlap was ambiguous. In this case we were able to successfully join the scaffolds with the help of an already available 1,211 bp willow warbler sequence from the mitochondrial control region (Bensch & Härlid 2000).

Annotation

Prior to annotation we used Repeatmodeler version 1.08 (Smit & Hubley 2015) to identify repeat sequences *de novo* in the assembly. The resulting repeat library was combined with other repeats from birds in the repeatmasker database version 20140131. The combined library was used with Repeatmasker version open-4.0.5 (Smit et al. 2015) to identify and mask repeats in the reference genome. The software identified 83 Mb (7.77%) of the assembly as repeats, with the most common type of repeats being Long Interspersed Nuclear Elements (LINEs).

Annotation of protein-coding genes was based on a combination of RNA sequence data, sequence homology to genes from other bird species and *ab initio* predictions. As RNA sequence data we made use of 1.8 million trimmed 454 cDNA reads originating from brain tissue of

several individuals from each subspecies (Lundberg et al. 2013). These were assembled into 29,022 transcripts using Newbler 2.6 (Margulies et al. 2005) with default settings for cdna assembly.

As homology evidence we included 16,354 transcript and protein sequences from 15,508 unique protein-coding genes in chicken, which were downloaded from ENSEMBL (www.ensembl.org) using Biomart, 1,388 transcripts from birds excluding chicken with a NM_ prefix from the RefSeq data base (http://www.ncbi.nlm.nih.gov/refseq/) and 5,856 proteins from birds excluding chicken from Uniprot (UniProt 2015) that were curated or supported by transcript or protein evidence.

All transcripts were mapped to the genome using GMAP 2014-09-30 (Wu & Watanabe 2005) using default settings with the exception of specifying a maximum intron size of 120 kb and cross-species alignments for other species than the willow warbler. Proteins were mapped using a combination of blastx and exonerate in MAKER version 2.31.6 (Holt & Yandell 2011). A preliminary set of gene models was created in MAKER by combining mapped evidence and gene predictions from augustus version 3.0.3 (Stanke et al. 2006) using the included parameters for chicken. We originally had problems with splitting of genes with longer introns. To avoid this problem for most of the large-intron genes, we used the 14,462 mapped transcripts from chicken as a first set of gene models. In a second run we updated the first set of gene models, and detected additional gene models, by using the rest of the evidence and gene predictions.

Genes that had been included in the first set of chicken transcript-based models were annotated based on their ENSEMBL id. The remainder of genes was annotated by blasting them to the full set of bird proteins used in MAKER (N=22,210) and to a set of 81,136 curated uniprot proteins from vertebrates excluding birds. In both cases, the best hit was determined by the

highest bitscore and with the condition that e-value should be less than 1e-10. For the bird proteins we also required a minimum of 60 % identity. Finally, we also used Interproscan 5 (Jones et al. 2014) for all models to assign functional domains. Gene models that were supported by prediction, but did not have any significant blast hit or assigned any functional domain, were discarded. The mitochondrial scaffold was annotated separately using the MITOS WebServer (Bernt et al. 2013), which detected 13 protein-coding genes, 2 rRNA and 22 tRNA.

Whole genome resequencing

Nine samples from each migratory phenotype were used for whole-genome resequencing (Table S1). In order to simplify analyses of genetic variation on the sex-linked Z chromosome only samples from male willow warblers were included. Samples were obtained from blood of birds that had been caught in mist nets. For a detailed description of catching and DNA extraction see Bensch *et al.*(1999). Quality control of samples prior to sequence library construction was the same as used for the *de novo* assembly. Sample-specific sequence libraries with a targeted insert size of 650 bp were prepared at ScilifeLab, Stockholm, Sweden, using a TruSeq DNA PCR free protocol (Illumina, CA, USA) with the same modifications as listed for the *de novo* sequencing. The libraries were multiplexed and sequenced on six lanes on a Hiseq2500 (Illumina, CA, USA) and subsequently quality-trimmed with the same pipeline as used for the *de novo* genome sequencing. One of the samples, which was the same used for *de novo* genome sequencing, produced very few reads. For this sample we decided to use the 650 bp library that had been included in the *de novo* assembly, which gave it a much higher coverage than the other samples (Table S1).

Reads were mapped to the genome assembly with *bwa* version 0.7.10 (Li & Durbin 2009) using the mem function and default settings except for -M flag, which was used for compatibility with downstream software. Alignments from different samples and lanes were mapped separately, but joined together into sample-specific bam files using the MergeSamFiles function of picardtools version 1.123 (http://broadinstitute.github.io/picard/). Once merged, duplicates were removed from the alignments using the MarkDuplicates function of picardtools.

Variant calling and filtering

We used freebayes version 0.9.21-8-g819db2b (Garrison & Marth 2012) to call genotypes from the filtered whole-genome resequencing data. Indel re-alignment and base quality recalibration on mapped reads, which are recommended steps prior to variant calling, are performed internally in the software. We used default settings with the exception of applying standard filters (minimum mapping and base quality of 30 and 20, respectively) and choosing not to report complex alleles (composites of indels and SNPs). The resulting variant calling format (vcf) file was first filtered using vcftools version 0.1.12b (Danecek et al. 2011) to exclude variants located in the repeat-masked regions, variants with a coverage double that of the mean coverage per site (>20x) and a quality less than 30, and genotypes with less than four supporting reads. For mean coverage calculation, and identification of SNPs exceeding this threshold, we temporarily excluded the sample that was used for the *de novo* sequencing due to its much higher coverage. Since population genetic estimates, such as genetic differentiation, could be misleading for a small number of genotypes, we included only variants with a maximum of two missing individuals in each of the migratory phenotypes. Finally, we left-aligned variants at each locus using the norm function in bcftools version 1.2 (Li 2011) in order to remove invariant positions

between alleles comprised of more than a single nucleotide. Variants on the mitochondrial scaffold were called separately be treating the input as haploid in freebayes. We noticed that we despite aggressive filtering obtained a very large number of SNPs (36 million). However, earlier sequencing studies have suggested that the willow warbler has an exceptionally high sequence diversity (Bensch et al. 2006b, Lundberg et al. 2011). In addition, we made further quality checks on the filtered SNP data to corroborate these results. The number of singletons per samples was relatively uniform, but as expected, showed an increase in samples with increased coverage (R=0.98, p=1.9E-11, excluding the reference sample 01L/19 which has much higher coverage). The SNP count in non-overlapping 10 kb windows was approximately normally distributed, with a mean and median of 340 and 365, respectively. This suggests that it is unlikely that the large number of SNPs is the result of some low-quality samples or large portions of the genome in which SNP calling is particularly challenging. Finally, the transition to transversion ratio across all SNPs was 2.09 and similar to what is expected across the human genome (Bainbridge et al. 2011).

Population genomics analyses

For the whole-genome resequencing data we estimated genetic differentiation and genetic diversity within and between subspecies for each bi-allelic SNP and over non-overlapping windows of 10 kb. Window-based estimates are influenced by the number of available sites that are covered by sequence data, and not taking this into account is likely to confound the analyses. In order to account for this, we quantified the number of callable sites for each window, i.e. those sites that fulfill the requirements for variant calling (minimum genotype depth=4, maximum two

missing genotypes in each population per site, minimum mapping quality=30 and minimum base quality=20). These filters were applied to the output of the samtools mpileup command and the subsequent number of callable positions per 10 kb window was calculated using the coverage command in bedtools version 2.17.0 (Quinlan & Hall 2010). For all measurements of genetic variation except F_{ST} we obtained window-based estimates by calculating the sum of per site estimates and dividing it by the number of callable sites for that window. Given the small number of re-sequenced samples, we requested that outlier windows outside should contain sufficient information (coverage and variants) to produce reliable inferences. For this purpose we required that each window should contain at least 5,000 callable sites, and in the case of F_{ST} also contain at least 25 SNPs. This filtering did not qualitatively affect the analysis, but removed noise.

Genetic differentiation was quantified as F_{ST} (Weir & Cockerham 1984) in vcftools, with a weighted average to represent 10kb windows. Following the approach of Roesti *et al.* (2012), we only included SNPs with MAF≥0.25 to avoid the down-biasing effect of the large number rare variants. This setting did not qualitatively change the results, but increased the contrast between the differentiated regions and the rest of the genome, and allowed for other, less divergent, regions to be detected.

For the mitochondrial scaffold we extracted haplotypes by superimposing variants of each sample to the mitochondrial reference sequence using the consensus command in bcftools and used DNASP version 5.10 (Rozas et al. 2003) to explore variation within and between the haplotypes from each migratory phenotype.

Geographical distribution of divergent region haplotypes

We used a multidimensional scaling (MDS)-based method in the R package invclust (Caceres & Gonzalez 2015) to determine if there was limited recombination between southern and northern haplotypes (i.e. haplotypes most common in either of the migratory phenotype) in each differentiated region. This method has been used to identify inversion polymorphisms, i.e. regions of reduced recombination between distinct groups of haplotypes, in humans and classify individuals as carriers of noninverted or inverted haplotypes. For this analysis, SNP genotypes within each differentiated chromosome region (chromosome 1: N=108, chromosome 3: N=86, chromosome 5: N=31) were analyzed jointly to provide coordinates of each sample along the two major axes of variation. With very restricted or absent recombination among the distinct groups of haplotypes, samples will fall into either of three equidistant clusters, with homozygote carriers of southern (SS) and northern (NN) haplotypes at each end, respectively, and heterozygous carriers (NS) clustering in the middle.

We further explored the change in haplotype frequencies of each region across the hybrid zones by fitting geographical clines using the R package HZAR (Derryberry et al. 2014). This was done by calculating the frequency of the northern haplotype at each sample site and assuming one-dimensional latitudinal clines across the hybrid zone in Sweden and one-dimensional longitudinal clines across the hybrid zone in Poland and Lithuania. For the differentiated region on chromosome 3, we did not fit any cline across the Polish/Lithuanian hybrid zone, because northern haplotypes were essentially lacking in this area. For each chromosome region we fit five different cline models, all with free scaling of allele frequency at the beginning and the end of the cline. The different models specified no exponential tails, exponential tail on the left or the right side, exponential tails mirrored on each side of the center

and independent tails on both side, respectively. Each model was fit to the data using 10^6 iterations of Markov Chain Monte Carlo (MCMC). The most probable model was chosen based on a corrected Akaike information criterion (AICc), which takes the number of parameters and observations into account. For the selected model, we extracted the center and the width of the cline and quantified the uncertainty of these estimates from parameters values within two likelihood units from the maximum likelihood.

We also examined whether the migratory phenotypes are more strongly associated with variation in any of the three differentiated regions, and more closely associated with these regions than other phenotypic traits (e.g. size measurements) that show some difference between the willow warbler subspecies. For this purpose we calculated the frequency of the northern haplotypes for each geographical sampling site and differentiated chromosome regions, as well as the sampling site mean of the following routinely measured phenotypic traits: the ratio of stable nitrogen isotopes in feathers (proxy for migratory phenotype), wing length, tarsus length, bill-head length and a color score (1-9) quantifying the whiteness on the breast relative to three reference specimens (see Bensch *et al.* (2009) for details). Because of the high correlation between the three length measurements (all in mm) we combined them into a single size measurement using the first principal component (explaining 58 % of the variation) from a PCA using the prcomp function in R and scaling of the variance of each measurement. We restricted the analyses to males (N=1,029, Table S3) as females differ from males in size measurements. The strength of the relationship between allele frequency and phenotypes was quantified using a Pearson correlation coefficient in R. These analyses are based on mean values per sampling sites rather than on the individual samples because the migratory trait (measured from feather stable isotopes) shows large individual variation within each migratory phenotype (Bensch et al.

2006a). However, this approach is accurate enough to assign a population sample to either of the two different African wintering areas, or to a population with either mixed or intermediate migration strategies (Bensch et al. 2009).

Breakpoint analyses and detection of structural variants

We used delly version 0.65 (Rausch et al. 2012) with default settings to identify structural variants from discordantly aligned read pairs and split reads. Inversion calls were further quality filtered using the delly script populationFilter.py.

As breakpoints could be located in between the ends of the regions and their neighboring scaffolds, we also designed primers that could amplify sequences across these gaps. In order to design suitable primers we extracted a few kb of scaffold sequence at each end of the divergent regions and sequences from the end of the adjacent scaffolds as determined from whole-genome alignments to the chicken, zebra finch and collared flycatcher genome. Since the scaffold order surrounding the region on chromosome 5 was much less clear than the other regions (Fig. S1), we focused our efforts on designing primers for the regions on chromosome 1 and 3. The scaffold ends were matched to other bird genomes on the ENSEMBL website (www.ensembl.org) using blastn with default settings. Matching sequences from the other bird genomes and the willow warbler scaffold ends were aligned to the zebra finch genome in Geneious (Drummond et al. 2012). Candidate primers (Table S9) were designed in particularly conserved parts of the alignments and blasted against the willow warbler genome to minimize the risk of designing primers in repetitive regions. Finally, we checked that the primer sites did not have any polymorphisms in the resequenced samples and that they had appropriate GC content and melting temperatures. For amplification we used a long-range PCR kit (Qiagen, CA,

USA) and followed the instructions of the manufacturer. PCR products were visualized on a 0.5 % agarose gel with 1kb ladder and lambda DNA for estimation of fragment sizes. For each locus, PCR products with the strongest band was extracted from the agarose gels using a Pasteur pipette, mixed with 100 µl of water and heated to 95ºC to dissolve the agarose. This was used as template for a second PCR and using a BigDye Terminator sequencing kit (Applied Biosystems, TX, USA).

Genetic variation within differentiated regions

The pattern of genetic variation within each group of northern and southern haplotypes combined with the genetic divergence between them provides additional information about the effect of selection, recombination rate and time since the haplotype groups separated. For example, reduced variation in these regions compared to the rest of the genome, could be indicative of low recombination rate, which would make the variation-reducing effect of background selection more pronounced. Similarly, low recombination rate could increase the magnitude of selective sweeps from positive selection. We divided the resequencing data into two sets of individuals that were either southern homozygous (N=8) or northern homozygous (N=6) for all of the three differentiated chromosome regions (Table S1). Twelve of the individuals in the resequencing data had been included in the SNP array and could be genotyped using the MDS analysis (see above). To obtain genotypes for all re-sequenced individuals we extracted genotypes of a subset of the MDS SNPs (N=174) that were also present in the filtered vcf file. We then added the genotypes from the array samples for the same subset of SNPs and performed the same MDS clustering as had been previously used. Before further analyses we checked that the clustering of this subset of data resulted in three distinct and equidistant groups, and that the twelve samples

that were included on both of the array and in the resequencing data had the same genotype. Once the re-sequence-based genotype data had been divided into groups of pure northern and southern individuals, we used vcftools to estimate nucleotide diversity and Tajima's D in each group for all bi-allelic SNPs within 10 kb windows. We used a customized perl script to calculate the average number of pairwise differences between sequences from each subspecies, d_{XY}, and the frequency of net number of substitutions, d_A, per site. Each of the site-specific estimates was summed over 10 kb windows and divided by the number of callable sites per window. For each divergent region we compared the window estimate distribution to that found across the rest of the genome excluding the other divergent regions.

As linked selection in parts of the genome with reduced recombination may give rise to large regions with increased relative differentiation such as F_{ST} (Cruickshank & Hahn 2014), we were particularly interested in comparing the position of the differentiated regions to the predicted centromere on the same chromosomes. For this purpose we made use of the whole-genome alignments of willow warbler scaffolds to the zebra finch genome and assumed that the centromeres would be located in a similar position in the genome of our focal species. The centromere on chromosome 5 was located on the unordered part of chromosome 5 (Tgu5_random), which was not included the whole-genome alignment analysis with the willow warbler genome. In this case we instead predicted the centromere interval by synteny to chromosome 5 in the chicken genome.

Functional annotation of differentiated regions

We further explored which genes and gene functions were associated with the individual variants and 10kb windows with the highest genetic differentiation. For this analysis we included

resequencing variants (SNPs and indels) with a F_{ST} of at least 0.7, the top 1 % most differentiated windows and array SNPs with a F_{ST} of at least 0.1. Individual variants were annotated by using SNPeff version 4.1l (Cingolani et al. 2012). Variants were assigned to one or several genes if they were within 5,000 bp upstream or downstream of them. For variants that were predicted to have a moderate to large functional impact on a gene, such as a missense mutations, we manually inspected the annotated gene models and their agreement with their supporting evidence in the Integrative Genome Viewer (IGV) software (Thorvaldsdottir et al. 2013). Windows were annotated using bedtools to intersect them with the annotated intervals of the genes

References

Amores, A., Catchen, J., Ferrara, A., Fontenot, Q. & Postlethwait, J. H. 2011. Genome evolution and meiotic maps by massively parallel DNA sequencing: spotted gar, an outgroup for the teleost genome duplication. *Genetics* **188**: 799-808.

Andrews, S. FastQC a quality-control tool for high-throughput sequence data. http://www.bioinformatics.babraham.ac.uk/projects/fastqc/.

Andrews, S. 2010. FastQC: a quality control tool for high throughput sequence data. Available online at: http://www.bioinformatics.babraham.ac.uk/projects/fastqc.

Bainbridge, M. N., Wang, M., Wu, Y., Newsham, I., Muzny, D. M., Jefferies, J. L., *et al.* 2011. Targeted enrichment beyond the consensus coding DNA sequence exome reveals exons with higher variant densities. *Genome Biol* **12**: R68.

Bensch, S., Andersson, T. & Åkesson, S. 1999. Morphological and molecular variation across a migratory divide in willow warblers Phylloscopus trochilus. *Evolution* **53**: 1925-1935.

Bensch, S., Bengtsson, G. & Åkesson, S. 2006a. Patterns of stable isotope signatures in willow warbler Phylloscopus trochilus feathers collected in Africa. *J Avian Biol* **37**: 323-330.

Bensch, S., Grahn, M., Müller, N., Gay, L. & Åkesson, S. 2009. Genetic, morphological, and feather isotope variation of migratory willow warblers show gradual divergence in a ring. *Mol Ecol* **18**: 3087-96.

Bensch, S. & Härlid, A. 2000. Mitochondrial genomic rearrangements in songbirds. *Mol Biol Evol* **17**: 107-13.

Bensch, S., Irwin, D. E., Irwin, J. H., Kvist, L. & Akesson, S. 2006b. Conflicting patterns of mitochondrial and nuclear DNA diversity in Phylloscopus warblers. *Mol Ecol* **15**: 161-71.

Bernt, M., Donath, A., Juhling, F., Externbrink, F., Florentz, C., Fritzsch, G., *et al.* 2013. MITOS: Improved de novo metazoan mitochondrial genome annotation. *Mol Phylogenet Evol* **69**: 313-319.

Bolger, A. M., Lohse, M. & Usadel, B. 2014. Trimmomatic: a flexible trimmer for Illumina sequence data. *Bioinformatics* **30**: 2114-20.

Borgstrom, E., Lundin, S. & Lundeberg, J. 2011. Large scale library generation for high throughput sequencing. *PLoS One* **6**: e19119.

Caceres, A. & Gonzalez, J. R. 2015. Following the footprints of polymorphic inversions on SNP data: from detection to association tests. *Nucleic Acids Res* **43**: e53.

Catchen, J., Hohenlohe, P. A., Bassham, S., Amores, A. & Cresko, W. A. 2013. Stacks: an analysis tool set for population genomics. *Mol Ecol* **22**: 3124-40.

Cingolani, P., Platts, A., Wang le, L., Coon, M., Nguyen, T., Wang, L., *et al.* 2012. A program for annotating and predicting the effects of single nucleotide polymorphisms, SnpEff:

SNPs in the genome of Drosophila melanogaster strain w1118; iso-2; iso-3. *Fly (Austin)* **6**: 80-92.

Cramp, S., ed. (1992) The birds of the Western Palearctic. Vol. VI. pp. Oxford University Press.

Cruickshank, T. E. & Hahn, M. W. 2014. Reanalysis suggests that genomic islands of speciation are due to reduced diversity, not reduced gene flow. *Mol Ecol* **23**: 3133-57.

Danecek, P., Auton, A., Abecasis, G., Albers, C. A., Banks, E., DePristo, M. A., *et al.* 2011. The variant call format and VCFtools. *Bioinformatics* **27**: 2156-8.

Derryberry, E. P., Derryberry, G. E., Maley, J. M. & Brumfield, R. T. 2014. HZAR: hybrid zone analysis using an R software package. *Mol Ecol Resour* **14**: 652-63.

Drummond, A. J., Ashton, B., Buxton, S., M., C., Cooper, A., Duran, C., *et al.* 2012. Geneious Pro 5.6.6. Available from: http://www.geneious.com/.

Etter, P. D., Bassham, S., Hohenlohe, P. A., Johnson, E. A. & Cresko, W. A. 2011. SNP discovery and genotyping for evolutionary genetics using RAD sequencing. *Methods Mol Biol* **772**: 157-78.

Garrison, E. & Marth, G. 2012. Haplotype-based variant detection from short-read sequencing. *arXiv* **1207.3907 [q-bio.GN]**

Gnerre, S., Maccallum, I., Przybylski, D., Ribeiro, F. J., Burton, J. N., Walker, B. J., *et al.* 2011. High-quality draft assemblies of mammalian genomes from massively parallel sequence data. *Proc Natl Acad Sci U S A* **108**: 1513-8.

Goudet, J. 2005. HIERFSTAT, a package for R to compute and test hierarchical F-statistics. *Mol Ecol Notes* **5**: 184-186.

Grabherr, M. G., Russell, P., Meyer, M., Mauceli, E., Alfoldi, J., Di Palma, F., *et al.* 2010. Genome-wide synteny through highly sensitive sequence alignment: Satsuma. *Bioinformatics* **26**: 1145-51.

Holt, C. & Yandell, M. 2011. MAKER2: an annotation pipeline and genome-database management tool for second-generation genome projects. *BMC Bioinformatics* **12**: 491.

Jones, P., Binns, D., Chang, H. Y., Fraser, M., Li, W., McAnulla, C., *et al.* 2014. InterProScan 5: genome-scale protein function classification. *Bioinformatics* **30**: 1236-40.

Kawakami, T., Smeds, L., Backström, N., Husby, A., Qvarnström, A., Mugal, C. F., *et al.* 2014. A high-density linkage map enables a second-generation collared flycatcher genome assembly and reveals the patterns of avian recombination rate variation and chromosomal evolution. *Mol Ecol* **23**: 4035-4058.

Laird, P. W., Zijderveld, A., Linders, K., Rudnicki, M. A., Jaenisch, R. & Berns, A. 1991. Simplified mammalian DNA isolation procedure. *Nucleic Acids Res* **19**: 4293.

Langmead, B. & Salzberg, S. L. 2012. Fast gapped-read alignment with Bowtie 2. *Nat Methods* **9**: 357-9.

Li, H. 2011. A statistical framework for SNP calling, mutation discovery, association mapping and population genetical parameter estimation from sequencing data. *Bioinformatics* **27**: 2987-2993.

Li, H. & Durbin, R. 2009. Fast and accurate short read alignment with Burrows-Wheeler transform. *Bioinformatics* **25**: 1754-60.

Li, H., Handsaker, B., Wysoker, A., Fennell, T., Ruan, J., Homer, N., *et al.* 2009. The Sequence Alignment/Map format and SAMtools. *Bioinformatics* **25**: 2078-2079.

Li, R., Zhu, H., Ruan, J., Qian, W., Fang, X., Shi, Z., *et al.* 2010. De novo assembly of human genomes with massively parallel short read sequencing. *Genome Res* **20**: 265-72.

Lindblad-Toh, K., Wade, C. M., Mikkelsen, T. S., Karlsson, E. K., Jaffe, D. B., Kamal, M., *et al.* 2005. Genome sequence, comparative analysis and haplotype structure of the domestic dog. *Nature* **438**: 803-19.

Lundberg, M., Åkesson, S. & Bensch, S. 2011. Characterisation of a divergent chromosome region in the willow warbler Phylloscopus trochilus using avian genomic resources. *J Evol Biol* **24**: 1241-1253.

Lundberg, M., Boss, J., Canbäck, B., Liedvogel, M., Larson, K. W., Grahn, M., *et al.* 2013. Characterisation of a transcriptome to find sequence differences between two differentially migrating subspecies of the willow warbler Phylloscopus trochilus. *BMC Genomics* **14**: 330.

Lundin, S., Stranneheim, H., Pettersson, E., Klevebring, D. & Lundeberg, J. 2010. Increased throughput by parallelization of library preparation for massive sequencing. *PLoS One* **5**: e10029.

Margulies, M., Egholm, M., Altman, W. E., Attiya, S., Bader, J. S., Bemben, L. A., *et al.* 2005. Genome sequencing in microfabricated high-density picolitre reactors. *Nature* **437**: 376-80.

Parra, G., Bradnam, K., Ning, Z., Keane, T. & Korf, I. 2009. Assessing the gene space in draft genomes. *Nucleic Acids Res* **37**: 289-97.

Patterson, N., Price, A. L. & Reich, D. 2006. Population structure and eigenanalysis. *PLoS Genet* **2**: e190.

Pigozzi, M. I. & Solari, A. J. 1998. Germ cell restriction and regular transmission of an accessory chromosome that mimics a sex body in the zebra finch, Taeniopygia guttata. *Chromosome Res* **6**: 105-13.

Purcell, S., Neale, B., Todd-Brown, K., Thomas, L., Ferreira, M. A., Bender, D., *et al.* 2007. PLINK: a tool set for whole-genome association and population-based linkage analyses. *Am J Hum Genet* **81**: 559-75.

Qing, H., Liu, G., Zhou, L., Wang, J., Li, L., Li, B., *et al.* 2015. Complete mitochondrial genome of Yellow-browed warbler Phylloscopus inornatus inornatus (Passeriformes: Sylviidae). *Mitochondrial DNA* **26**: 939-40.

Quinlan, A. R. & Hall, I. M. 2010. BEDTools: a flexible suite of utilities for comparing genomic features. *Bioinformatics* **26**: 841-2.

Rausch, T., Zichner, T., Schlattl, A., Stutz, A. M., Benes, V. & Korbel, J. O. 2012. DELLY: structural variant discovery by integrated paired-end and split-read analysis. *Bioinformatics* **28**: I333-I339.

Roesti, M., Salzburger, W. & Berner, D. 2012. Uninformative polymorphisms bias genome scans for signatures of selection. *BMC Evol Biol* **12**: 94.

Rousset, F. 2008. genepop'007: a complete re-implementation of the genepop software for Windows and Linux. *Mol Ecol Resour* **8**: 103-6.

Rozas, J., Sanchez-DelBarrio, J. C., Messeguer, X. & Rozas, R. 2003. DnaSP, DNA polymorphism analyses by the coalescent and other methods. *Bioinformatics* **19**: 2496-7.

Sambrook, J., Fritsch, E. F. & Maniatis, T. 1989. *Molecular cloning, a laboratory manual. Vol. 2.* Cold Spring Harbor Laboratory Press, Cold Spring Harbor, NY.

Smit, A. & Hubley, R. 2015. RepeatModeler Open, http://www.repeatmasker.org

Smit, A., Hubley, R. & Green, P. 2015. RepeatMasker Open-4.0, http://www.repeatmasker.org.

Stanke, M., Schoffmann, O., Morgenstern, B. & Waack, S. 2006. Gene prediction in eukaryotes with a generalized hidden Markov model that uses hints from external sources. *BMC Bioinformatics* **7**: 62.

Thorvaldsdottir, H., Robinson, J. T. & Mesirov, J. P. 2013. Integrative Genomics Viewer (IGV): high-performance genomics data visualization and exploration. *Brief Bioinform* **14**: 178-92.

UniProt, C. 2015. UniProt: a hub for protein information. *Nucleic Acids Res* **43**: D204-12.

Warren, W. C., Clayton, D. F., Ellegren, H., Arnold, A. P., Hillier, L. W., Kunstner, A., *et al*. 2010. The genome of a songbird. *Nature* **464**: 757-762.

Weir, B. S. & Cockerham, C. C. 1984. Estimating F-Statistics for the Analysis of Population-Structure. *Evolution* **38**: 1358-1370.

Wu, T. D. & Watanabe, C. K. 2005. GMAP: a genomic mapping and alignment program for mRNA and EST sequences. *Bioinformatics* **21**: 1859-75.
